# Effect of Sulfonated Inorganic Additives Incorporated Hybrid Composite Polymer Membranes on Enhancing the Performance of Microbial Fuel Cells

**DOI:** 10.3390/polym15051294

**Published:** 2023-03-03

**Authors:** Gowthami Palanisamy, Sadhasivam Thangarasu, Tae Hwan Oh

**Affiliations:** School of Chemical Engineering, Yeungnam University, Gyeongsan 38541, Republic of Korea

**Keywords:** microbial fuel cell, bioenergy, water treatment, membrane, separator, hybrid polymer composite, sulfonation, additives

## Abstract

Microbial fuel cells (MFCs) provide considerable benefits in the energy and environmental sectors for producing bioenergy during bioremediation. Recently, new hybrid composite membranes with inorganic additives have been considered for MFC application to replace the high cost of commercial membranes and improve the performances of cost-effective polymers, such as MFC membranes. The homogeneous impregnation of inorganic additives in the polymer matrix effectively enhances the physicochemical, thermal, and mechanical stabilities and prevents the crossover of substrate and oxygen through polymer membranes. However, the typical incorporation of inorganic additives in the membrane decreases the proton conductivity and ion exchange capacity. In this critical review, we systematically explained the impact of sulfonated inorganic additives (such as (sulfonated) sSiO_2_, sTiO_2_, sFe_3_O_4_, and s-graphene oxide) on different kinds of hybrid polymers (such as PFSA, PVDF, SPEEK, SPAEK, SSEBS, and PBI) membrane for MFC applications. The membrane mechanism and interaction between the polymers and sulfonated inorganic additives are explained. The impact of sulfonated inorganic additives on polymer membranes is highlighted based on the physicochemical, mechanical, and MFC performances. The core understandings in this review can provide vital direction for future development.

## 1. Introduction

As the world’s population grows, resulting in the overconsumption of energy, mostly from fossil fuels, it has a detrimental influence on the environment through green gas emissions, resulting in global warming and a changing climate and biosphere emergency [1,2,3]. We need cleaner energy technology to solve this problem as soon as possible. This includes saving energy by making it more efficient, using fewer fossil fuels, and making environment-friendly energy sources more accessible. Accordingly, renewable energy sources, such as water, sun, wind, biomass, and geothermal technologies, have been addressed as an alternative to conventional energy sources [4,5,6]. Therefore, scientists worldwide have come up with different ways to convert and store electrochemical energy, such as fuel cells [7,8,9], batteries [10,11], and electrochemical capacitors [12,13]. Electrochemical energy conversion and storage technologies have been considered an alternative due to their eco-friendly and sustainable routes [14]. Over the past several decades, fuel cell technologies have gained considerable attention because of their enhanced energy conversion efficiency, energy density, and reliability, and they work continuously until there is an endless supply of fuel and oxygen [8,15,16]. Based on the electrolyte, fuels, and operating temperature, the fuel cells are classified into proton exchange membrane fuel cells [17], alkaline fuel cells [18], solid oxide fuel cells [19], phosphoric acid fuel cells [20], microbial fuel cells (MFCs) [21,22,23], enzymatic fuel cells [24], direct methanol fuel cells [25], molten-carbonate fuel cells [26], direct ethanol fuel cell [27], unitized regenerative fuel cells [28,29], direct borohydride fuel cells [30], direct ethylene glycol fuel cells [31], direct glycerol fuel cells [32], and direct formic acid fuel cells [8,33].

**Figure 1:**
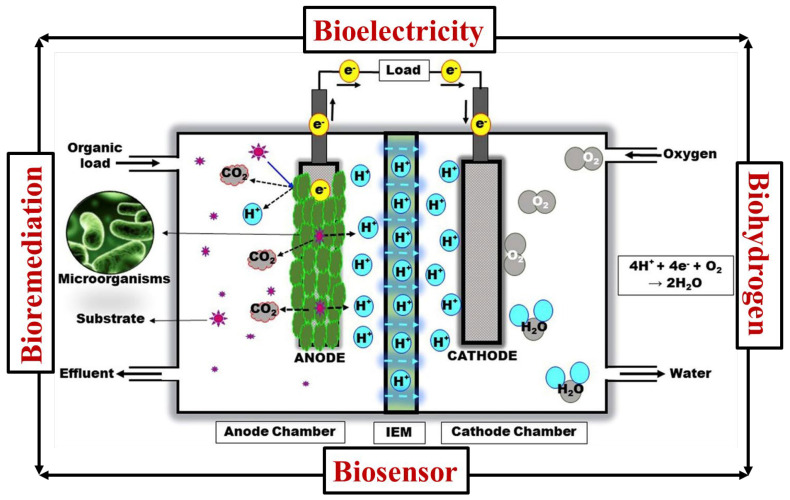
A schematic representation of dual-chambered microbial fuel systems reaction process and applications. Reproduced from [34], with permission from Elsevier, 2019.

Among the different fuel cell technologies, the MFC is considered an excellent energy system [34,35,36,37]. The most exciting advantage of MFCs is generating electricity via biological microorganisms using wastewater [38,39,40], such as industrial wastewater [41,42,43]. Generally, the MFC generates electricity using organic waste [44,45]. The MFC has been widely considered in various sectors, such as environmental science, engineering, electrochemistry, and energy [46,47]. In terms of application, an MFC has been widely believed to produce biohydrogen, bioelectricity, and biosensors and also be used in bioremediation processes [48,49,50,51]. During MFC operation, MFC allows for simultaneous bioenergy production and bioremediation in a single device. The MFC usually emits carbon dioxide during unit cell operation. However, the MFC system was carbon-neutral for generating energy [52,53,54]. Based on the unit cell alignments, the MFCs have been classified into different categories, such as stacked, up-flow, double-chamber, and single-chamber MFCs [55,56]. Figure 1 [34] depicts the dual-chamber MFC process schematically. Here, the anode chamber and the cathode chamber have been separated by the ion exchange membranes [57,58,59]. In MFCs, the center part of the ion exchange membrane has a predominant role during the unit cell operation, such as ion transportation, controlling the substrate crossover, and prohibiting the oxygen crossover [60,61,62,63,64]. The functioning of electron transportation and ion transportation in MFCs is similar to the proton exchange membrane fuel cell [65,66]. In MFCs, electricity is commonly derived using microbial sources. In MFCs, the microorganism breaks down organic matter to make electrons, protons, and carbon dioxide as byproducts [67,68]. As shown in Figure 1, an electron is transferred to the cathode using an external circuit, and protons are transported to the cathode using a proton exchange membrane from the anode side to the cathode side. At the cathode, oxygen, protons, and electrons combine to form water, a byproduct of an oxygen reduction reaction (Figure 1). Some examples of oxidation of organic matter through microorganisms in an anode chamber are as follows [69,70,71]:

Sucrose: C_12_H_22_O_11_ + 13H_2_O → 48H^+^ + 48e^−^ + 12CO_2_

Glucose: C_6_H_12_O_6_ + 6H_2_O → 24H^+^ + 24e^−^ + 6CO_2_

The example of cathode chamber reaction is as follows:

4H^+^ + 4e^−^ + O_2_ → 2H_2_O

The main things that affect the performance of MFCs are the components, the process variables, and the way the unit cells are set up [40,72,73]. In MFCs, the proton exchange membrane is one of the most critical components to attaining excellent performance for generating electricity and wastewater treatment [74,75,76]. Perfluorosulfonic-acid-based commercial membranes (e.g., Nafion and CMI 7000) have been used as an ideal proton exchange membrane candidate for MFC applications because of their proton exchange capability and stability [77,78,79,80,81]. Commercial membranes have certain drawbacks, such as a high production cost, high oxygen/substrate crossover, biological accumulation, biofouling, and deprivations [82,83]. Various kinds of polymer membranes have been developed in different categories, such as composite membranes, blend membranes, reinforced membranes, sandwich-type membranes, and crosslinked membranes [84,85,86,87,88,89,90,91,92], to overcome these issues.

Among the various approaches, incorporating inorganic nanomaterials in the polymer matrix to create a hybrid composite membrane is an important consideration for the MFC. Inorganic nanomaterials are frequently used in membranes to improve their dimensional, mechanical, thermal, and chemical properties. In MFCs, the inorganic nanoparticles are used to control the fuel crossover and change the physicochemical properties of the membrane, in addition to making it more stable. The incorporated inorganic nanomaterial fillers are classified into various categories, such as inert hygroscopic, proton conductor, and proton-conducting properties with hydrophilic nature [93,94,95]. In most cases, the inorganic nanofillers control the membrane’s fuel crossover, dimensional stability, and mechanical strength [96,97,98,99]. However, incorporating inorganic nanofillers can significantly affect proton transport behavior in most membranes, which is a disadvantage of inorganic nanofillers. To improve the efficacy of inorganic nanofillers, surface modification and functionalization have been executed for different kinds of inorganic nanofillers and utilized for MFC applications. Among the various changes and functionalization, introducing the sulfonic acid functional group (-SO_3_H) in the inorganic nanomaterials is an excellent way to promote proton conductivity and decrease the fuel crossover in the membrane. The sulfonic acid functional group enhances the hydrophilicity of the membrane and also holds the membrane’s dimensional stability, which is a significant benefit of using sulfonated inorganic additives. In addition, the sulfonation site in the inorganic fillers makes it possible for hydrogen bonds to form between polymers and inorganic materials. The membrane contains higher dimensional stability due to its hydrophilic nature. An acid functional group can effectively transfer the protons from the anode to the cathode via the vehicular (diffusion) and Grotthuss (hopping) mechanisms. This phenomenon generates more ionic channels and carriers for efficient proton transport. As a result, many different kinds of inorganic additives have been tried for MFC applications. The sulfonation of nanoparticles (NPs) and nanomaterials has positively tuned the properties, such as water uptake (WU), dimensional stability, ion exchange capacity (IEC), proton conductivity (PC), biofouling, and mechanical stability and performance of membranes, for MFC applications. This review systematically explained the impact of nonsulfonated and sulfonated inorganic additives on different kinds of polymers for MFC applications. According to the developments of sulfonated inorganic additives for MFC applications, the evaluation is described in five sections; namely, sulfonated silica incorporated polymer membrane, sulfonated TiO_2_ containing polymer membrane, sulfonated graphene oxide containing polymer membrane, sulfonated Fe_3_O_4_ containing polymer membrane, and different kinds of sulfonated additives in polymer membrane.

## 2. Sulfonated Silica Incorporated Polymer Membrane for MFC Applications

Inorganic silica (SiO_2_) has recently been widely regarded as an excellent nanofiller material in membranes for various energy devices [100,101,102,103]. Generally, the inorganic SiO_2_ contains considerable properties for membranes, such as an increased surface area with excellent physical, chemical, and thermal properties [104]. Moreover, the incorporation of inorganic SiO_2_ into the polymer matrix enhances the PC of the composite membrane due to the high water-holding property of SiO_2_ [105]. Different ideas about SiO_2_ have been made and studied in this context for use in MFC membranes. Kircheva et al. investigated the MFC performance of a low-cost microporous polyethylene (PE)/silica (PE-Si) membrane in an air-cathode MFC, finding that the PE-Si membrane performed similarly to the Nafion 117 membrane under different conditions using nonparametric analysis (Mann–Whitney) [106]. In another approach, composite membranes have been developed by SiO_2_-polyvinyl chloride (PVC) by solution casting method [107]. The PC of the composite membrane has been increased by incorporating hygroscopic silica gel due to its high WU property and the inclusion of the desired ratio of citric acid and phosphotungstic acid (PWA). Increasing the concentration of silica increases the IEC and WU capacities. Though particle agglomeration has increased the silica concentration beyond the limit, this has decreased the mechanical strength. The maximum power density (PD) obtained was 43.91 mW cm^−2^ for the composite membrane containing 5% silica with 5% PWA [107]. 

**Figure 2:**
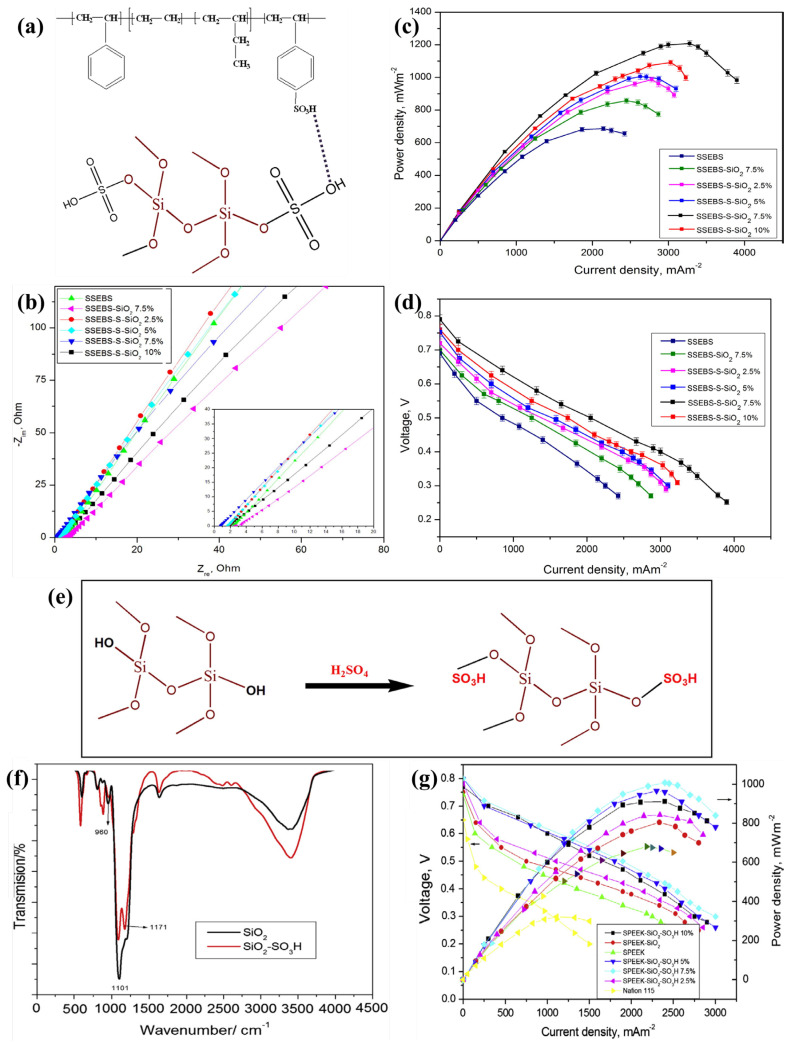
(**a**) A graphical representation of SSEBS polymer and sSiO_2_ inorganic filler interaction. (**b**) Nyquist plots, (**c**) power density, and (**d**) polarization curves of SSEBS, SSEBS/SiO_2_ (7.5%), and SSEBS/sSiO_2_ (2.5, 5, 7.5, and 10%) membranes. Reproduced from [104], with permission from Elsevier, 2016. (**e**) Preparation of sSiO_2_, (**f**) FT-IR of SiO_2_ and sSiO_2_, and (**g**) polarization curve and power density of Nafion 115, SPEEK, SPEEK-SiO_2_, and SPEEK-sSiO_2_ membranes. Reproduced from [105], with permission from Elsevier, 2015.

Angioni et al. introduced mesoporous silica (SBA-15) and modified it with propylsulphonic groups (10 and 50 mol.% of SBA-SO_3_H) as fillers into the Nafion membrane [108]. The prolonged study of MFCs using wastewater showed that the Nafion-SBA15−SO_3_H (10 mol.%) membrane exhibited higher PD (380 mW m^−3^) than the Nafion membrane with higher COD removal efficiency (95%) and enhanced coulombic efficiency (CE) (34%). Moreover, SBA15-SO_3_H-based fillers protect the surface of the composite membrane from biofouling. Thus, the mesoporous SBA-SO_3_H fillers in Nafion could be used to develop composite membranes for MFC wastewater treatment [108]. Incorporating inorganic additives enhances the PC of the composite membrane at high temperatures [104]. In case of low temperature (30 °C), the PC of the composite membrane has been reduced [104,109,110]. Hence, sulfonation of the inorganic additives has been incorporated to increase PC at low temperatures. In this way, Sivasankaran et al. fabricated a PEM comprising sulfonated SiO_2_ (sSiO_2_—2.5, 5, 7.5, and 10%) into sulfonated polystyrene ethylene butylene polystyrene (SSEBS) for single-chamber MFC (SCMFC) [104]. The interaction between sSiO_2_ and SSEBS is shown in Figure 2a. By increasing the s-SiO_2_ content in the composite membrane, the IEC (3.015 meq/g) and PC (0.321 S/cm) (Figure 2b) increased up to 7.5% s-SiO_2_ concentration. In similar conditions, the IEC and PC of pure SSEBS are 1.825 meq/g and 1.52 × 10^−2^ S/cm, respectively. It makes the s-SiO_2_ concentration even higher (over 7.5%) and decreases the PC of the membrane because too much s-SiO_2_ causes it to clump together. Because of this, the composite membrane’s ability to take in water worsens, making it harder for protons to pass through membranes. Due to its higher PC and lower oxygen permeability, the SSEBS-s-SiO_2_ 7.5% composite membrane has been found to improve the performance of the SCMFC (Figure 2c,d); 7.5% sSiO_2_ in the SSEBS composite membrane resulted in a higher PD of 1209 ± 17 mW m^−2^ [104]. In another study, Sivasankaran and Sangeetha prepared (Figure 2e,f) a sSiO_2_-containing composite membrane with SPEEK [105] instead of SSEBS polymer for SCMFC. The presence of sSiO_2_ in the SPEEK polymer enhanced the WU, IEC, and PC of the SPEEK-sSiO_2_ membrane. Therefore, MFCs with a SPEEK-sSiO_2_ membrane generated a power output of 1008 mW m^−2^ (Figure 2g), three times more than Nafion 115 (320 mW m^−2^). Moreover, the SPEEK-sSiO_2_ membrane revealed excellent unit cell performance compared to the SPEEK (680 mW m^−2^) and SPEEK-SiO_2_ (nonsulfonated SiO_2_-802 mW m^−2^) membranes [105]. Moreover, the same research group investigated the effect of a SPEEK-SiO_2_ nanocomposite membrane on microbial community via tubular MFC in generating electricity by treating sewage wastewater [111]. It was also observed that sulfonated inorganic filler increased the PC of composite membrane because the sulfonated organic–inorganic materials in the membrane enhanced the performance [111]. Table 1 presents the physical and chemical properties and evaluation results of sulfonated silica incorporated polymer membranes for MFC applications.

## 3. Sulfonated TiO_2_ Containing Polymer Membrane for MFC Applications

The inorganic nanofillers of TiO_2_ and its modified version gained potential advantages for developing organic–inorganic hybrid composite membranes due to their hygroscopic, chemical properties and inbuilt antibacterial effect [112,113]. Membrane fouling has been reduced in the composite membrane containing TiO_2_ due to its hydrophilic properties, which increase the membrane surface hydrophilicity, thus decreasing the hydrophobic interaction between microorganisms and the membrane surface [114]. Christophe Pagnout et al. studied the electrostatic interaction between *Escherichia coli* and TiO_2_. In this case, at neutral pH, the TiO_2_ does not show any toxicity toward *E.coli*. Still, it exhibits toxicity in acidic and alkaline pH conditions due to the positive and negative charges of TiO_2_ and bacterium, respectively [115]. Introducing TiO_2_ and sulfonated TiO_2_ (sTiO_2_) provides considerable benefits for developing the hybrid membrane for various energy systems [116,117,118,119,120]. Bajestani and Mousavi developed a Nafion-TiO_2_-1 wt% membrane using different solvents (NMP, DMAc, and DMF) [112]. The characteristic membrane behaviors were investigated along with the impact of casting solvents. The solvents used in membrane preparation are responsible for the membrane morphology through their volatility and polymer–solvent–NP interaction. Nafion-TiO_2_ with DMF demonstrated strong hydrogen bonding interactions and higher porosity among the tested solvents. Thus, the Nafion-TiO_2_ in the DMF solvent membrane showed higher WU (51%), PC (0.0126 S/cm), and IEC (1.32 meqg^−1^). Moreover, the enhanced PC of Nafion-TiO_2_ with DMF solvent exhibits an increased OCV (330 mV) of MFCs at steady-state conditions [112].

Sulfonated polyether ether ketone (SPEEK) is an excellent candidate among hydrocarbon polymers due to its low cost and higher chemical and mechanical stability [121,122]. The degree of sulfonation in SPEEK is proportional to the membrane’s PC [120]. However, the increased amount of -SO_3_H functional group in the SPEEK membrane increased the WU and swelling ratio, reducing the membrane’s mechanical stability [122]. To address the issues, various inorganic fillers, including TiO_2_, were introduced to modify the properties of SPEEK membranes [123]. In this connection, Venkatesan and Dharmalingam prepared a SPEEK-TiO_2_ composite membrane using the rutile structure of TiO_2_ for MFC applications [124]. An increased oxygen mass transfer coefficient and cation transport behavior were observed for the composite membrane. Among different concentrations of TiO_2_ (2.5, 5.0, 7.5, and 10%), the 7.5% TiO_2_ in SPEEK composite membrane showed efficient IEC, PC, antifouling properties, and PD. It has been seen that increased TiO_2_ content (10% TiO_2_) exhibits a blocking effect and controls the ionic channels, which affects the PC of the membrane [124]. In another approach, different ratios of TiO_2_ (2.5, 5, 7.5, and 10%) in SPEEK membranes were developed for generating electricity from dairy wastewater [125]. Among different ratios, SPEEK with a 5% TiO_2_ composite membrane showed increased WU (31%) and IEC (1.71 meqg^−1^). From the MFC performances, it has been identified that SPEEK with a 5% TiO_2_ composite membrane showed increased PD (1.22 Wm^−2^) and voltage (0.635 V). Moreover, the hybrid composite membrane exhibits a high COD removal efficiency [125]. The Ti-based compounds of perovskite oxides, such as strontium titanate (SrTiO_3_), have also been used as filler for their high hydrophilic nature that enhances the WU property [126,127,128]. By means of this, silica particles can improve the homogenous dispersivity of the polymer matrix. When surface-modified nanofillers are added to the polymer matrix, they make the membrane stronger than when unmodified nanofillers are added. Bhowmick et al. incorporated the modified SrTiO_3_/Si and TiO_2_/Si and nanofillers into the PVA polymer matrix for fabricating PEM [126]. The base polymer PVA possesses high hydrophilic properties and film-forming ability due to the cross-linking of the available –OH groups. SrTiO_3_/Si-PVA and TiO_2_/Si-PVA composite membranes generated PDs of 5.39 ± 0.27 and 6.16 ± 0.31 Wm^−3^, respectively. The induced PDs were comparable to Nafion and 30 times less expensive for developing the composite membrane [126]. Considering cellophane as the base material for membrane developments provides an alternative option for generating electricity and pollutant removal using MFCs. In this case, a 1:1 ratio mixed combination of ZnO and TiO_2_ NPs (0.03%) was incorporated in the cellophane and further altered with the bio-inspired polydopamine [129]. This combination has been effectively considered because of its novel, cost-effective, antibiofouling, and self-cleaning properties. Power generation was increased during MFC operation by increasing cation transport during the oxidation process via PDA modifications and catalyst mixing of the modified membrane. In addition to this, the membrane surface fouling and lifespan were reduced by the increased hydrophilic property of membrane modifications [129].

**Figure 3:**
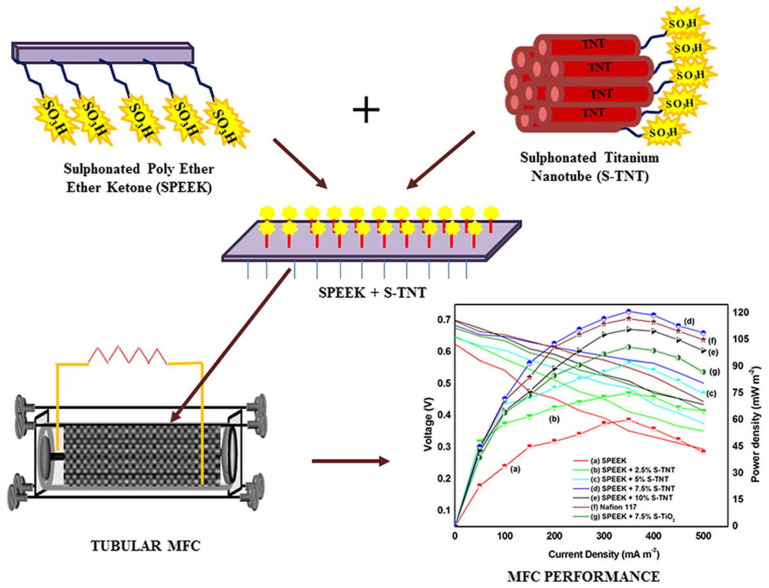
A schematic representation of sulfonated PEEK and STiO_2_ nanotube composite membrane developments for tubular MFC and MFC performances (polarization curves and power density) of sPEEK, SPEEK with different ratios of STiO_2_ nanotube (2.5, 5, 7.5, and 10%) membranes. Reproduced from [130], with permission from Elsevier, 2020.

**Figure 4:**
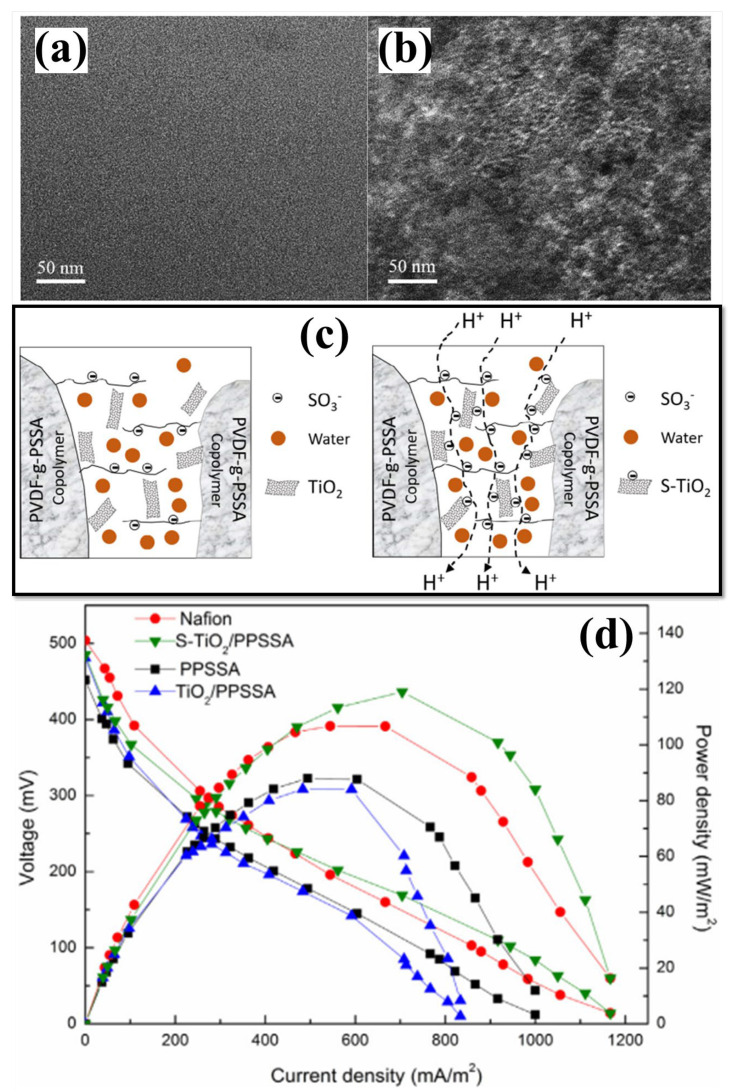
Transmission electron microscope images of (**a**) PPSSA and (**b**) PPSSA-sTiO_2_ membranes. (**c**) Diagrammatic representation of the PPSSA-sTiO_2_ membranes’ proton transport routes. (**d**) Polarization and power density curves of Nafion, PPSSA, PPSSA-TiO_2_, and PPSSA-sTiO_2_ membranes. Reproduced from [139], with permission from Elsevier, 2019.

Its surface properties have been modified in multiple ways to enhance the efficiency of TiO_2_ NPs in membranes. Besides creating more proton transport channels, the surface modification of TiO_2_, primarily sulfonation, also acts as a proton carrier vehicle owing to its negative charge [130,131]. However, due to the neutral charge, metal oxides (-OH group) and water molecules generated proton transport pathways [131]. In addition, the hydrophilic sulfonic acid groups exchanged the hydroxyl group of TiO_2_ during sulphonation that further enhancing the antibiofouling characteristic of the composite membrane [132]. In this view, the impact of sulfonated TiO_2_ (sTiO_2_) has been evaluated using a SPEEK polymer membrane [133]. The sTiO_2_ in the composite enhances PC and reduces oxygen diffusion. The results showed that MFCs with SPEEK-sTiO_2_ (7.5 wt.%) membrane generated an excellent PD of 1202.5 mW m^−2^ with a reduced internal resistance of 37 Ω [133]. The same research group prepared a novel SPEEK-based composite membrane with hydrothermally synthesized sulfonated titanium nanotube (S-TNT) for enhancing the membrane lifespan, PC and antibiofouling properties for electricity generation through tubular MFC (Figure 3) [130]. The incorporated hollow S-TNT has a high water-holding capacity that helps to increase the proton transport mechanisms. By using tubular MFC (300 mL), the maximum PD of 121 mWm^−2^ (with 79.37% COD removal) was obtained for the SPEEK/S-TNT (7.5%) composite membrane. The higher performance is mainly possessed by a high IEC value of 3.2 meqg^−1^ with decreased internal resistance (30 Ω) [130]. Another approach was made with novel sulfonated polystyrene–ethylene–butylene–polystyrene and sulfonated TiO_2_ (SPSEBS/sTiO_2_) nanocomposite membranes as PEM for MFC applications [131]. The membrane PC was enhanced by sulfonating the inorganic TiO_2_ nanofiller and delivered the highest PD of 1345 ± 17 mW m^−2^ for SPSEBS/STiO_2_ 7.5%, which was found to be 124% higher than MFCs with pristine SPSEBS membrane [131]. For evaluating MFC performance, a combination of SPSEBS polymer with various percentages of synthesized S-TNT was used, as well as statistical optimization (Box–Behnken factorial design) of operational parameters (catalyst concentration, external resistance, and substrate type) [134]. Here, the STNT in the composite membrane improves hydrophilicity, mechanical stability, and PC with the highest antibiofouling property in MFC application [134]. Cost-effective polyvinylidene fluoride (PVDF) polymers have been considered a membrane candidate for MFC applications. PVDF is also considered a base polymer material for preparing PEM due to its better thermal and chemical properties [135,136,137]. To enhance the PC of PVDF, modifying PVDF through a polymer grafting method in which sodium styrene sulfonate directly reacts with ozone-preactivated PVDF powder and is grafted into leading chains resulted in the development of PVDF-g-PSSA membrane with enhanced mechanical and chemical stability with PC [138]. Although the hydrophobic nature of PVDF causes membrane biofouling on the membrane surface in PVDF-g-PSSA membranes, this makes them not suitable for long-term operation in MFCs [138]. Hence, Li et al. created sTiO_2_ by grafting sulfonic groups onto TiO_2_ and embedding it in PVDF-g-PSSA membranes to create a sTiO_2_-PSSA composite membrane with enhanced PC and antibiofouling properties using a solution casting technique [139]. Transmission electron microscopy (TEM) analysis (Figure 4a,b) has shown that PPSSA and sTiO_2_-PPSSA have both hydrophilic and hydrophobic regions and ionic aggregation. The hydrophilic region refers to darker regions, and the hydrophobic region refers to brighter areas. Here, the hydroxyl groups in the inorganic TiO_2_ filler have been substituted with a sulfonic acid group in sTiO_2_. Therefore, adding of sTiO_2_ increased the hydrophilicity of the STiO_2_-PSSA composite membrane, which made the membrane better at absorbing water. As a result of the interdependent effect of Grotthuss and vehicle mechanisms, the development of proton transport pathways increases membrane PC (Figure 4c). The sTiO_2_-PSSA composite membrane stability and the antibiofouling property have been evaluated through MFC operation for 2 months with their obtained PDs and polarization curves (Figure 4d). The fouled composite membrane demonstrated an excellent PD and a 91% higher COD removal efficiency than the Nafion 117 membrane [139]. Table 2 displays the physicochemical properties and performance evaluation results of polymer membranes with different kinds of TiO_2_ added for MFC applications.

## 4. Sulfonated Graphene Oxide Containing Polymer Membrane for MFC Applications

The two-dimensional structure of graphene oxide (GO) is used as an inorganic filler during composite membrane fabrication for its excellent physicochemical characteristics, such as high hydrophilicity, PC, more surface area, and tremendous mechanical strength [140,141,142]. The presence of different oxygen functional groups (i.e., hydroxyl, carboxylic, and epoxy groups) in GO is responsible for its enhanced hydrophilicity. The rigid mechanical strength of GO is also due to the strong covalent bonds in its hydrophobic region [140,142]. As discussed, the SPEEK polymer exhibits immense PEM properties, such as excellent thermal, mechanical, chemical stability, hydrophilicity, and low cost with high ionic conductivity. In this view, Leong et al. successfully fabricated a GO/SPEEK composite PEM with self-synthesized GO as a nanofiller [140]. GO/SPEEK composite membranes are enhanced for transferring protons through the Grotthuss mechanism by a hydrogen bonding network that increases the composite membrane PC. Thus, the GO-SPEEK composite membrane exhibits enhanced physicochemical properties with improved PC (1.48 × 10^−3^ S cm^−1^) and oxygen diffusion coefficient (1.154 × 10^−6^ cm^2^ s^−1^). The performance of MFCs with GO-SPEEK composite membrane exhibited a higher CE (16.88%) than Nafion 117 (12.31%) and was recommended as a promising alternative to Nafion 117 membrane in MFCs [140]. A similar kind of composite PEM was developed by Shabani et al. by introducing nano-sized GO into SPEEK for generating electricity while simultaneously treating wastewater [143]. In MFCs, the SPEEK-GO composite membrane outperformed the commercial Nafion 117 membrane in terms of WU, PD (53.12 mW m^−2^), and CE (3.74 0.18%), as well as COD removal efficiency (88.71 0.29%) [143]. In another approach, GO-SPEEK and Silver-GO/GO/SPEEK (AgGO-GO-SPEEK) self-fabricated composite membranes were developed and used in MFC applications for their higher PC, reduced oxygen crossover, and good antibiofouling properties [144]. The inclusion of Ag NPs inhibits microbial growth in the membrane. The AgGO-GO-SPEEK membrane had a 54.2% higher PC and a 76.7% lower oxygen diffusion coefficient than the commercial Nafion 117 membrane. Furthermore, the AgGO-GO-SPEEK composite membrane demonstrated low internal resistance with improved PD during MFC operation and outperformed the commercial Nafion 117 membrane [144]. Along with SPEEK polymer, other sulfonated hydrocarbon polymers (e.g., sulfonated polyethersulfone (SPES), sulfonated polysulfone) have sparked interest in composite PEMs. Ali et al. developed a GO-incorporated SPES composite membrane (GO/SPES) in MFCs for simultaneous electricity generation and wastewater treatment [145]. The prepared GO-SPES composite membrane exhibited a higher PD of 101.2 mW m^−2^ and a current density of 613 mA m^−2^ with an 80% COD removal efficiency [145]. Inexpensive and high-availability PVA with higher thermal and chemical stability was also introduced as a polymer matrix for developing PEM. Introducing the inorganic fillers into the PVA polymer matrix enhances its PC property and tunes its physical and electrochemical properties to be suitable for IEM. GO, an inorganic filler, was impregnated into a PVA and silicotungstic acid (STA) matrix for developing a low-cost PVA/STA/GO composite membrane by solution casting technique for generating electricity using SCMFCs [146]. The GO incorporation in the composite membrane enhances its PC and antibiofouling properties. Moreover, SCMFCs with a PVA/STA/GO composite membrane generated a maximum PD of 1.9 W m^−3^ by treating acetate wastewater with 91% substrate removal efficiency [146]. Likewise, Rudra et al. prepared a graphite-oxide-based nanocomposite membrane for harvesting electricity from SCMFCs containing crosslinked PVA and sulfonated styrene (SS) (polymerized in situ) as the backbone matrix [147]. The WU, swelling behavior, and oxygen diffusivity of the composite membrane have been reduced due to the hydrogen bonding of graphite oxide to the substrate. Thus, the composite membrane with 0.4% graphite oxide in SCMFCs exhibited a higher PD of 193.6 mW m^−2^ with 803.33 mA m^−2^ current density and ~81.89% COD removal efficiency [147].

Recently, novel PEM has been developed by incorporating GO nanofiller into PVDF and cellulose acetate (CA) polymer blend and used in MFCs for generating clean energy [148]. The presence of CA in this composite membrane has improved its ability to stop biofouling, the conductivity of protons, porosity, and the ability to attract water. In the same way, the thermal and chemical stability of the composite membrane was attributed to PVDF and GO for improving surface area along with membrane hydrophilicity [148]. This study was extended by the same group, in which superior low-cost PEM was developed, consisting of PVDF, CA, and reduced GO (rGO) for extracting electricity using MFCs [137]. During MFC performances, it was seen that the performance of the composite membrane was similar to that of commercial membranes. It has been seen that composite membranes generated a higher COD removal efficiency (92.0 ± 0.8%) along with 118 mW m^−2^ of PD [137]. Despite developing composite PEM using a synthetic polymer matrix, Holder et al. utilized biopolymers such as chitosan (CS), in which GO was introduced as a filler to form CS/GO composite membrane [149]. In addition, CS/GO cross-linked with sulfuric acid or phosphoric acid for developing CS/GO-P and CS/GO-S composite membranes. During MFC operation using wastewater, CS/GO-P-membrane-equipped MFCs generated maximum PD (16.35 mW m^−3^) and COD removal efficiency (89.52%). In the same conditions, the CS/GO-S delivered a PD of 6.94 mW m^−3^ and COD removal efficiency of 31.99% [149].

**Figure 5:**
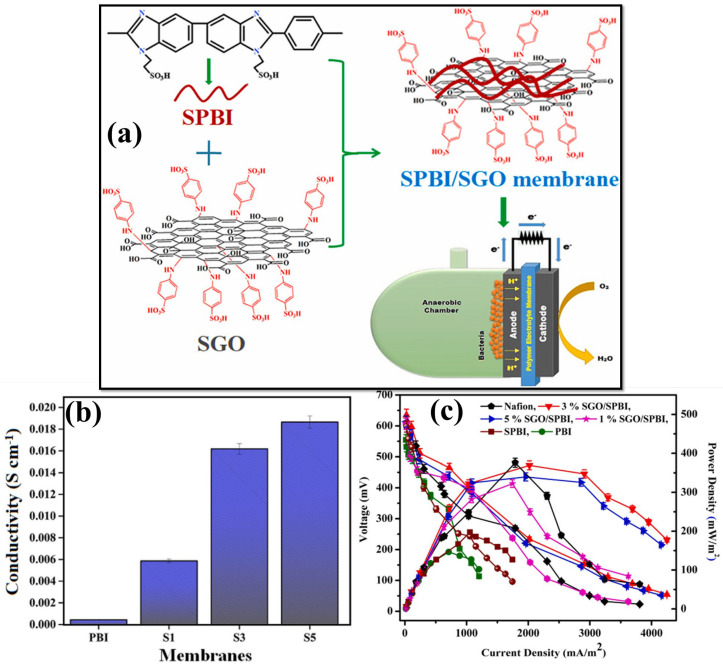
(**a**) A schematic representation of SPBI-SGO membrane preparation progression for MFC system. (**b**) Proton conductivity and (**c**) MFC performances of PBI, SPBI, and different concentrations of SGO (1, 3, and 5%) in the SPBI membrane. Reproduced from [150], with permission from Elsevier, 2020.

**Figure 6:**
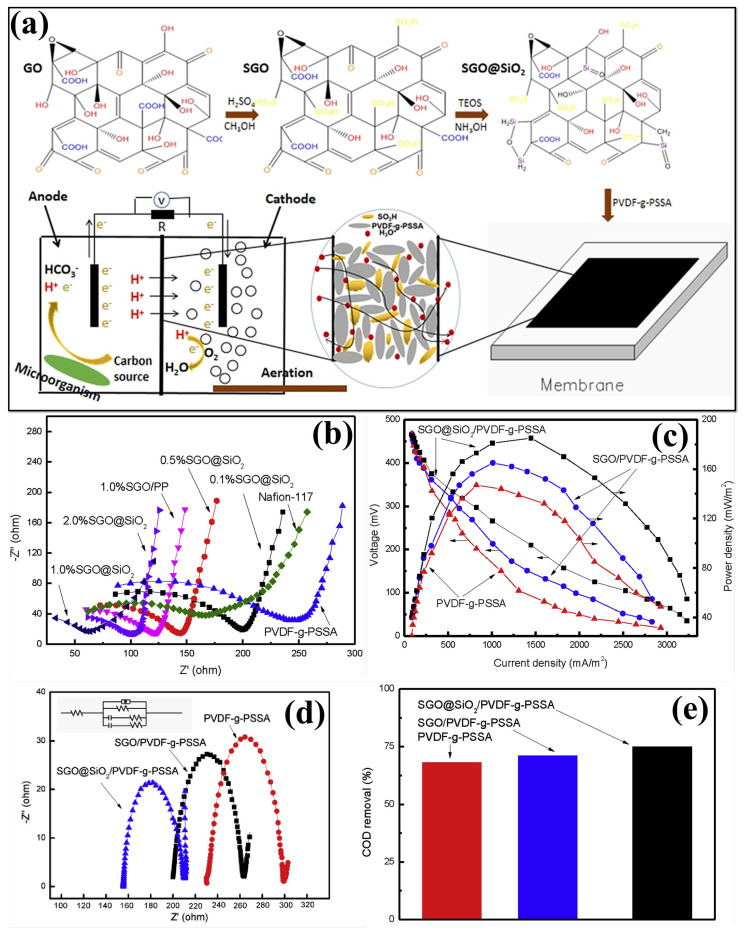
(**a**) A schematic representation of SGO@SiO_2_ preparation steps and PVDF-g-PSSA/SGO@SiO_2_ membrane developments for MFC device. (**b**) EIS performance of Nafion 117, PVDF-g-PSSA, and different ratios of PVDF-g-PSSA/SGO@SiO_2_ membranes. (**c**) Polarization curves, (**d**) EIS, and (**e**) COD removal of PVDF-g-PSSA, PVDF-g-PSSA/SGO and PVDF-g-PSSA/SGO@SiO2 membranes. Reproduced from [155], with permission from Elsevier, 2019.

Because of a reasonable amount of oxygen-containing functional groups (e.g., hydroxyl, carboxyl, and -SO_3_H groups), sulfonated GO (sGO) can effectively increase the PC, mechanical stability, and antifouling properties in the hybrid composite membrane when compared to GO [150,151,152]. The phosphoric-acid-doped PBI can also be used as a base polymer matrix for its excellent chemical, thermal, and mechanical properties to develop composite membranes for MFC applications [89,150,153]. The PC depends on an acid-doped membrane’s acid doping level (ADL). However, increasing the ADL in the membrane reduces its mechanical strength. Hence, modifications such as incorporating inorganic nanofillers have been performed to develop PBI nanocomposite with enhanced mechanical and thermal stability and dimensional stability. In this way, Mondal et al. fabricated sulfonated SPBI-based composite PEM by introducing different proportions of SGO (Figure 5a) [150]. For sulfonating PBI polymers, it reacts with 2-chloro ethane sulfonic acid, in which the PBI imidazole nitrogen atom has been covalently grafted with alkyl sulfonic acid. The GO nanofiller was synthesized using a modified Hummers’ method and sulfonated with chlorosulfonic acid. Figure 5b shows that SPBI-5% SGO exhibited a higher PC of 0.018 S cm^−1^ than pristine PBI membrane (4.28 × 10^−4^ S cm^−1^). This increment was attributed to the development of more proton transport channels through H-bonding developed by a higher content of –SO_3_H groups in the PBI matrix during SGO inorganic filler incorporation. During the MFC operation (Figure 5c), it was observed that the homogenous and widespread distribution of SGO in the 3% SPBI-SGO composite membrane exhibited a higher OCV of ~669 ± 18 mV and a maximum current density of 2019.06 mA m^−2^ at 0.234 V. It was expected that MFCs with a 5% SPBI-SGO composite membrane would exhibit a higher OCV and current density due to its enhanced PC and IEC values. However, it performed worse than the 3% SPBI-SGO composite membrane, owing to a higher concentration of SGO agglomerate on the PBI surface and more sulfonic acid groups on the membrane surface. Therefore, the substrate-generated cations interacted with those groups enhancing membrane resistivity. It was also noted that the maximum PD of 472.46 mW m^−2^ was generated by MFCs equipped with 3% SPBI-SGO than pristine PBI, SPBI, or other composite membranes. Hence, MFCs with a 3% SPBI-SGO composite membrane exhibited increased current generation with reduced voltage drops, recommended as an alternative PEM candidate for MFC application [150]. Moreover, Shabani et al. prepared a cost-effective sulfonated polyethersulfone (SPES)-based hybrid composite membrane by introducing GO, thiolated GO (TGO), and SGO filler for enhancing MFC performances [154]. The proton selectivity of the composite membrane was improved by introducing -SO_3_H and sulfhydryl (-SH) groups in the fillers. From this, the 1.8% SPES-SGO composite membrane exhibited higher proton (H^+^) selectivity than other cations (Li^+^, N^+^, and K^+^). Hence, the functionalized GO nanocomposite membranes in MFCs showed higher PD and CE with higher COD removal efficiency [154]. A novel composite antibiofouling PEM has been developed with the introduction of SGO into the PVDF-g-PSSA polymer matrix, and its MFC performance was evaluated [151]. The SO_3_^−^ groups in the composite PVDF-g-PSSA/SGO membrane increase the WU, hydrophilicity, and ionic conductivity of the membrane, which enhances the electricity generation during MFC operation [151]. In another way, the same research group made new PVDF-g-PSSA-based composite membranes with SiO_2_-filled SGO nanofillers (Figure 6a) to improve the PC and antibiofouling properties [155]. SiO_2_ has been inserted into SGO through in situ hydrolysis, with ethyl orthosilicate used as a precursor to obtain SiO_2_/SGO. Furthermore, the various concentrations of developed SiO_2_/SGO NPs (0.1, 0.5, 1.0, and 2.0%) are prepared and added to the PVDF-g-PSSA polymer matrix. Increasing the -SO_3_H content of the membrane improves PC, as seen in the impedance spectra in Figure 6b. Incorporating SiO_2_/SGO enhances the proton transport channels for migration and increases the composite membrane’s hydrophilic properties (WU%). These improvements improved the composite membrane’s PC, which was higher at 1% SiO_2_/SGO. Further increasing the SiO_2_/SGO NP content decreases the composite membrane’s PC values through the particle agglomeration that prevents the polymer chain from free movement and thus reduces the proton transport. MFCs with PVDF-g-PSSA/SGO/SiO_2_ membrane outperformed MFCs in terms of PD and current density (185 mW m^−2^ and 1338 mA m^−2^, respectively) as shown in Figure 6c. In addition, low resistance and reduced membrane fouling result in 75% COD removal efficiency (Figure 6d,e) [155]. The physicochemical properties and performance evaluation results of sulfonated GO-incorporated polymer membranes for MFC applications have been provided in Table 3.

## 5. Sulfonated Fe_3_O_4_ Containing Polymer Membrane for MFC Applications

**Figure 7:**
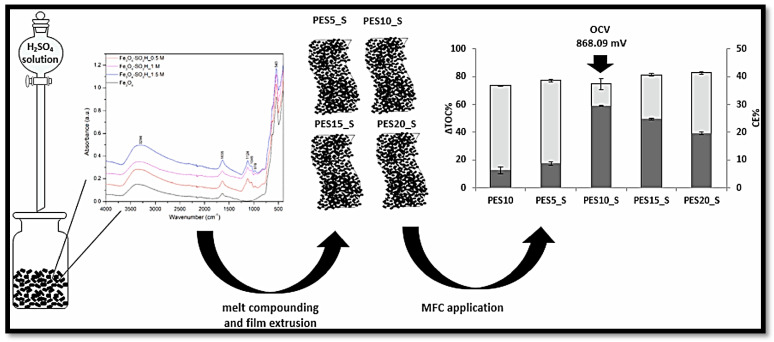
The overall process (FT-IR spectra of Fe_3_O_4_ and sFe_3_O_4_) and performances (coulombic efficiency (grey) and total organic carbon removal) of sulfonated Fe_3_O_4_/PES nanocomposite membranes. Reproduced from [162], with permission from Elsevier, 2020.

Developing hybrid membranes with Fe_3_O_4_ and its modified version for various systems was intriguing [156,157,158]. In this connection, Prabhu and Sangeetha prepared the composite SPEEK-Fe_3_O_4_ by incorporating different weight percentages (2.5, 5.0, 7.5, and 10%) of Fe_3_O_4_ into the SPEEK polymer matrix [159]. The effect of Fe_3_O_4_ on the composite membrane was measured in terms of its electrochemical performance, ability to take in water, ability to let oxygen pass through it, and ability to conduct ions. The 7.5% Fe_3_O_4_-SPEEK composite membrane fabricated MFCs exhibited a lower oxygen diffusion rate with increased PC and attained a maximum PD of 104 mW m^−2^ [159]. Solution casting techniques are commonly preferred for developing polymer nanocomposite membranes because of their efficiency and applicability, even in smaller quantities [81,160]. However, this technique is unsuitable during large-scale membrane development for its innate inappropriateness over commercial standards. Palma et al. fabricated a polyethersulfone (PES)-based nanocomposite membrane using different contents of Fe_3_O_4_ NPs using melt extrusion membrane for developing the membrane on a large scale [81]. The composite membrane performance was evaluated using H-type MFC with sodium-acetate-containing synthetic wastewater. Incorporating Fe_3_O_4_ NPs into the membrane increases its mechanical strength, which is desirable for long-term stable MFC operation. During MFC operation, the highest PD of 9.59 ± 1.18 mW m^−2^ and current density of 38.38 ± 4.73 mA m^−2^ for composite membrane with 20% Fe_3_O_4_ NPs incorporation were reported [81]. In a second study, they looked at how pretreating PES/Fe_3_O_4_ nanocomposite membranes affected the electrochemical performance of MFCs [161]. It was observed that pretreating the membrane involved boiling the composite membrane for 1 h in deionized water and immersing it in H_2_SO_4_ (0.5 M) for 1 h. As a result, PES-Fe_3_O_4_ (10 wt%) composite membrane exhibited a membrane resistance of 7.55 kΩ with a PD of 10.59 ± 0.72 mW m^−2^ and a current density of 52.07 ± 0.86 mA m^−2^. Hence, the pretreatment method reduces the incorporation of the NPs used effectively for MFC applications [161]. In another approach, permanently modified magnetite NPs filler (Fe_3_O_4_) has been achieved through the direct degree of sulfonation using various amounts of sulfuric acid (0.5, 1, and 1.5 M) [162]. The micro- and crystalline nature of the magnetite NPs structure was not altered during the sulfonation process, as confirmed by experimental analysis. Then, the composite PES-based membrane was fabricated by introducing various contents of sFe_3_O_4_ (5, 10, 15, and 20 wt%), and their performance was evaluated in H-type MFC using an acetate substrate. Here, the large number of –SO_3_H and -OH functional groups enhances the membrane hydrophilicity, which improved the WU, IEC, and PC values with reduced oxygen diffusion properties of the sulfonated membranes. The absorption peaks for pristine Fe_3_O_4_ and sulfonated Fe_3_O_4_ NPs were analyzed through the FTIR technique and shown in Figure 7, which is used to confirm the sulfonation functional group. During 350 h of MFC operation, the MFC coupled with the PES20_S composite membrane exhibited the highest total organic carbon (TOC) removal rate of 82.74% (Figure 7). It was observed that the PES10_S composite membrane showed the highest power and current densities of 270% and 117% higher than the Nafion 117 membrane, respectively, along with 868.09 mV of OCV and 29.58% of CE [162]. The physicochemical properties and performance evaluation results of sulfonated Fe_3_O_4_ incorporated polymer membranes for MFC applications have been provided in Table 4.

## 6. Different Kinds of Sulfonated Additives in Polymer Membrane for MFC Applications

The outstanding chemical stability, conductivity, and antimicrobial property of silver (Ag) were also investigated for its application as an inorganic filler in fabricating composite PEM for MFC applications [144,167,168,169]. The antimicrobial activity of Ag not only reduces the biofilm formation in the membrane but also reduces the microbial growth responsible for generating electrons [167,170,171]. Thus, the optimizing Ag NPs concentration as fillers in composite membrane preparation is necessary. Kugarajah and Dharmalingam fabricated a SPEEK-Ag composite membrane by incorporating different concentrations of Ag into the SPEEK polymer matrix and studying their effect on MFC performance and the microbial community [167]. During MFC operation, a maximum PD of 156 ± 0.5 mW m^−2^ was achieved for SPEEK-7.5 wt% Ag than other composite membranes or Nafion 117. Furthermore, the antibiofouling effect of Ag in composite membranes contributed to its long-term operation and improved MFC performance [167]. Tiwari et al. utilized cost-effective borosilicate glass as a backbone matrix incorporated with PVA/Nafion for casting a PVA/Nafion/borosilicate composite (MPN) membrane for MFC applications [172]. It was observed that MFCs with MPN membrane generated a higher PD (6.8 W m^−3^) than Nafion 117 membrane (7.1 W m^−3^). Hence, the low-cost MPN membranes are an alternative to the Nafion 117 membrane in real-time MFC applications [172]. Apart from inorganic fillers, heteropoly acids (HPAs) such as silico tungstic acids (STAs) with high protonic conductivity range (2 × 10^−2^ S cm^−1^) have also been used as fillers in polymer matrix for improving the stabilities and PC [173]. The hybrid composite membrane was fabricated by entrapping STA particles in the SPEEK membrane for SCMFC applications. The SPEEK-7.5% STA composite membrane exhibited a high power density (207 mW m^−2^) than Nafion 117 during SCMFC operation [173]. Similarly, phosphotungstic acid (PWA) and sulfonated polystyrene (SPS) have been introduced into the SPEEK polymer matrix to create more proton pathways for enhanced PC in a developed composite PEM for MFC applications [174]. Saniei et al. created hydrophilic goethite NPs and their derivatives, goethite–tannic acid–sulfanilic acid NPs, and goethite–tannic acid NPs [175]. The composite SPEEK membrane has been fabricated by embedding the as-prepared NPs into the SPEEK polymer matrix and is used as a membrane in MFC operations. SPEEK with 0.5 wt% of goethite exhibited higher PD (73.7 mW m^−2^), current density (293 mA m^−2^), and CE (52.6%) with increased COD removal efficiency (97.6%) [175]. Recently, Sugumar and Dharmalingam prepared a new PEM by solution-casting sulfonated polyhedral oligomeric silica (S-POSS) into the SPSEBS base polymer matrix to improve mechanical strength and PC [163]. They also statistically optimized (RSM) various pH, substrate concentrations, and anode materials with SPSEBS/6%-SPOS composite membranes for generating electricity through tubular MFCs. It has been observed that maximum PD (126 mW m^−2^) was obtained at 75% substrate concentration, maintaining the pH 7 with graphite rod as anode material in MFCs operating over 3 weeks [163]. The same research group took a different approach, incorporating S-POSS into the SPEEK polymer matrix to create a promising PEM candidate for improving tubular MFC stability and performance [164]. The inorganic S-POSS filler created more ion transport channels in the composite membrane with its hydroxide ions. This made the PC and WU go up. Along with this, they observed that bacterial strains such as *Firmicuteswere*, *Gammaproteobacteria*, and *Betaproteobacteria* identified as bioelectrogenesis and provided the future view on different electron transfer mechanisms [164]. The physicochemical properties and performance evaluation results of various sulfonated additives incorporated in polymer membranes for MFC applications have been provided in Table 4.

**Figure 8:**
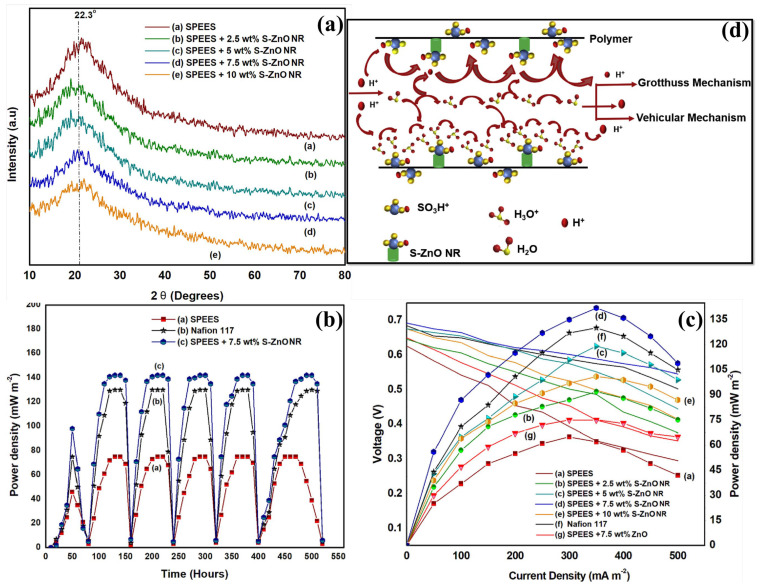
(**a**) XRD spectra of SPEES and different concentrations of sZnO NR (2.5, 5, 7.5, and 10%). MFC performances of (**b**) power density vs. time and (**c**) voltage vs. current density. (**d**) A graphic illustration of the ion transport mechanism in SPEES-sZnO NR composite membrane. Reproduced from [165], with permission from Elsevier, 2021.

In some instances, zinc oxide (ZnO) is used in anode or cathode catalysts for enhancing antibiofouling properties by suppressing the formation of biofilms [165,176,177]. In the case of membrane applications as nanofillers, ZnO NPs are converted into nanorods (ZnO NR) to increase the surface area for enhancing the WU property [165]. If ZnO NR fillers were used in the composite membrane, the ionic conductivity was attributed solely to the vehicular mechanism (via water). As a result, sulfonation of ZnO NR (S-ZnO NR) is performed in addition to the vehicular mechanism to increase PC via Grotthuss mechanism (via SO_3_H groups), as shown in Figure 8d. As a result, the researchers incorporated different sulfonated ZnO NR (hydrothermally synthesized) ratios (2.5, 5, 7.5, and 10%) into a sulfonated poly(phenylene ether ether sulfone) (SPEES) polymer for fabricating SPEES/SZnO NR composite membranes in tubular MFCs. At this point, the SPEES polymer and ZnO NR filler enhance the membrane’s hydrophilic properties, improving its water holding, IEC, and PC. Both vehicular (through water) and Grotthuss mechanism (through -SO_3_H) means of proton transport have been observed in SPEES/SZnO NR composite membranes, due to the presence of nanorod structure (improving WU property) and the sulfonic acid group. In this case, ionic transport is also aided by SO_3_^−^ domains that exhibit strong electrostatic attraction, with the -SO_3_H group interacting directly with the polymer structure (Figure 8d). In addition, S-ZnO NR incorporation also improves the composite membrane’s mechanical stability and antibiofouling property. At the start of an MFC operation, the voltage output goes down because the uptake of microorganisms means the medium needs to be filled with new material. It was observed (Figure 8c) that MFCs equipped with SPEES/7.5% SZnO NR delivered a maximum PD of 142 ± 1.2 mW m^−2^ with lower internal resistance and was recommended to be used as PEM in MFCs for a long time of operation [165]. In a different approach, the same research group incorporated SZnO into SPSEBS polymer to prepare PEM, which was then used in MFCs for the green electricity generation [166]. Here, SZnO NR was used to improve the proton transport mechanisms and make the proton transport more conductive. This made the surface area bigger and the material more water-friendly. The highest PC of 1.49 × 10^−2^ S cm^−1^ has been observed for the SPSEBS/6% SZnO composite membrane [166]. Thus, it has been confirmed that the homogeneous addition of inorganic additives to the polymer matrix effectively improved the overall physicochemical properties, stabilities, and MFC performances.

## 7. Concluding Remarks

Recently, global researchers have raised significant concerns for the energy and environmental research and development sectors. In this case, energy generation and water remediation are urgently important categories to address for a safer human life. Among the various devices, the MFC can produce bioenergy by cleaning wastewater. Thus, considerable attention has been provided to the MFC system. Usually, the MFC’s performance depends on components and process variables. The proton exchange membrane is the most important candidate for making the MFC work best by controlling the substrate/oxygen crossover and improving proton transport. There are different kinds of commercial membranes available for MFC applications. However, commercial membranes have certain disadvantages, such as high cost and fuel crossover. To overcome these issues in the polymer membrane, different kinds of inorganic additives have been included homogeneously. The inclusion of inorganic additives has significantly improved the membrane properties and performance. This review details the impact of sulfonated inorganic additives on their functional group surface properties and the base polymer material. Sulfonated inorganic additives, such as sulfonated silica (S-SiO_2_), sulfonated titania (S-TiO_2_), sulfonated graphene oxide (S-GO), sulfonated iron oxide (S-Fe_3_O_4_), and sulfonated zinc oxide (S-ZnO), have effectively altered the overall physicochemical properties of the membrane. Furthermore, the surface morphology of the inorganic additive (such as nanoparticles, nanotubes, 2D nanosheets, and core@shell) plays an important role in the membrane for controlling the oxygen crossover. The sulfonic acid functional group enhances the hydrophilicity of the polymer membrane and also holds the membrane’s dimensional stability, which are the significant benefits of using sulfonated inorganic additives. The membrane contains higher dimensional stability with hydrophilic nature, and an acid functional group can effectively transfer the protons from the anode to the cathode via the vehicular (diffusion) mechanism and Grotthuss (hopping) mechanism. This phenomenon generates more ionic channels and carriers for efficient proton transport. Thus, the overall performance of MFC-containing hybrid (organic polymers and inorganic-sulfonated additives) composite membranes has been higher than that of commercial membranes and corresponding pure polymer membranes. To further improve the overall performance of membrane for MFC applications, numerous efforts can be made by developing the inorganic additives with various concepts: (i) dimensional modifications (e.g., developing 2D-structured inorganic additives containing proton exchange site will be more effective in controlling the crossover of oxygen without affecting the proton transport behavior); (ii) functionalization of different properties of inorganic additives (Very limited inorganic additives have been studied as a nanofiller for MFC membranes. Therefore, excellent inorganic additives can be identified using various options with different functionalization.); and (iii) combinations with different kinds of organometallics and inorganic polymers.

## Figures and Tables

**Table 1:** Physicochemical properties and performance evaluation results of sulfonated silica incorporated polymer membrane for MFC applications.

IA	Polymer	WU (%)	IEC(meqg^−1^)	PC S/cm)	K0 (cms^−1^)	IR (Ω)	PD (mWm^−2^)	CE (%)	Ref.
	Nafion			0.085					[108]
Mesoporous silica (SBA-15)	Nafion			0.028					
SBA-SO_3_H10	Nafion			0.062				34	
SBA-SO_3_H50	Nafion			0.088					
	SSEBS	164 ± 7	1.825		3.5 × 10^−5^	66	680 ± 13	75	[104]
SiO_2_-7.5%	SSEBS	185 ± 8	1.622		0.9 × 10^−5^	60	852 ± 11	78	
S-SiO_2_-2.5%	SSEBS	175 ± 10	1.990		0.98 × 10^−5^	55		82	
S-SiO_2_-5%	SSEBS	187 ± 9	2.55		0.8 × 10^−5^	45		80	
S-SiO_2_-7.5%	SSEBS	210 ± 8	3.015	3.21 × 10^−2^	0.75 × 10^−5^	37	1209 ± 17	85	
S-SiO_2_-10%	SSEBS	200 ± 11	2.821		0.67 × 10^−5^	39		83	
	Nafion	22	0.982	2 × 10^−2^	1.6 × 10^−4^		290 ± 7		
	SPEEK	23	1.12		3 × 10^−6^	71 ± 3	680	75 ± 4	[105]
SiO_2_-5%	SPEEK	39	1.41		0.9 × 10^−6^	62 ± 2	802	79 ± 4	
S-SiO_2_-2.5%	SPEEK	35	1.65		1.2 × 10^−6^	55 ± 2	864	85 ± 5	
S-SiO_2_-5%	SPEEK	40	1.73		1 × 10^−6^	50 ± 1	912	87 ± 6	
S-SiO_2_-7.5%	SPEEK	42	1.80	1.018 × 10^−2^	0.84 × 10^−6^	46 ± 0.5	1008 ± 17	90 ± 7	
S-SiO_2_-10%	SPEEK	37	1.74		0.7 × 10^−6^	52 ± 1	810	85 ± 6	
		22		3.0 × 10^−2^	1.6 × 10^−5^		320 ± 6		
	SPEEK	37 ± 0.3	1.15 ± 0.06	0.96 × 10^−2^	2.2 × 10^−6^	39	59 ± 1.2		[111]
2.5wt% S-SiO_2_	SPEEK	38 ± 0.5	1.34 ± 0.03	0.99 × 10^−2^	1.74 × 10^−6^	37	72 ± 1.6		
5wt% S-SiO_2_	SPEEK	39 ± 0.4	1.54 ± 0.05	1.14 × 10^−2^	1.64 × 10^−6^	32			
7.5wt% S-SiO_2_	SPEEK	40 ± 0.6	1.82 ± 0.08	1.24 × 10^−2^	1.42 × 10^−6^	29	154 ± 1.5		
10 wt% S-SiO_2_	SPEEK	39 ± 0.3	1.71 ± 0.04	1.1 × 10^−2^	1.49 × 10^−6^	34			
	Nafion	22 ± 0.4	1.2 ± 0.05	0.85 × 10^−2^	2.42 × 10^−6^	45	145 ± 1.6		

IA—inorganic additives, WU—water uptake, IEC—ion exchange capacity, PC—proton conductivity, K0—oxygen mass transfer coefficient, IR—internal resistance, PD—power density, CE—coulombic efficiency.

**Table 2:** Physicochemical properties and performance evaluation results of sulfonated TiO_2_ containing polymer membrane for MFC applications.

IA	Polymer	WU (%)	IEC (meqg^−1^)	PC S/cm)	K0 (cms^−1^)	IR (Ω)	PD (mWm^−2^)	CE (%)	COD (%)	Ref.
S-TiO_2_-5%	SPEEK	37	0.99		0.7 × 10^−6^					[133]
TiO_2_-5%	SPEEK	25	0.75		0.7 × 10^−6^					
	SPEEK	20	0.9		3 × 10^−6^					
S-TiO_2_-2.5%	SPEEK	32	0.95		0.5 × 10^−6^					
S-TiO_2_-7.5%	SPEEK	39	1.05	1.382 × 10^−2^	0.8 × 10^−6^	37	1202.5			
S-TiO_2_-10%	SPEEK	38	0.94		0.87 × 10^−6^					
	Nafion	22		0.30 × 10^−2^	1.6 × 10^−5^	125	300			
	SPEEK	37.2	1.8	0.97 × 10^−2^	2.2 × 10^−6^	39	59			[130]
S-TNT-2.5%	SPEEK	37.9	2.5	1.1 × 10^−2^	1.74 × 10^−6^	35	70			
S-TNT-5%	SPEEK	38.5	2.8	1.24 × 10^−2^	1.54 × 10^−6^	33				
S-TNT-7.5%	SPEEK	39	3.2	1.37 × 10^−2^	1.32 × 10^−6^	30	121	51 ± 2	79.37	
S-TNT-10%	SPEEK	38.2	2.9	1.0 × 10^−2^	1.49 × 10^−6^	37				
	Nafion	22	1.2	0.81 × 10^−2^	2.4 × 10^−6^	45	117			
S-TiO_2_-7.5%	SPEEK	37.6	2.5	1.18 × 10^−2^	1.42 × 10^−6^	35	102			
	SPSEBS	163 ± 3	1.89	1.52 × 10^−2^	3.5 × 10^−5^	66 ± 4	695 ± 7	75 ± 3		[131]
TiO_2_-7.5%	SPSEBS	170 ± 5	1.62	1.08 × 10^−2^	0.8 × 10^−5^	60 ± 3	835 ± 8	72 ± 2		
S-TiO_2_-2.5%	SPSEBS	185 ± 8	2.25	1.75 × 10^−2^	0.8 8 × 10^−5^	55 ± 2	975 ± 11	80 ± 4		
S-TiO_2_-5%	SPSEBS	200 ± 9	3.02	2.51 × 10^−2^	0.7 × 10^−5^	45 ± 1	1200 ± 15	83 ± 3		
S-TiO_2_-7.5%	SPSEBS	220 ± 11	3.35	3.57 × 10^−2^	0.64 × 10^−5^	35 ± 0.8	1345 ± 17	87 ± 4		
S-TiO_2_-10%	SPSEBS	218 ± 10	3.02	2.72 × 10^−2^	0.60 × 10^−5^	37 ± 0.5	1105 ± 13	85 ± 5		
	PVDF-g-PSSA	25 ± 0.2		0.046 ± 0.003		243.95	106.67		85	[139]
TiO_2_-1%	PVDF-g-PSSA	28.3 ± 0.3		0.041 ± 0.002		309.67	98.18		86	
S-TiO_2_-1%	PVDF-g-PSSA	33.2 ± 0.2		0.048 ± 0.003						
S-TiO_2_-2.5%	PVDF-g-PSSA	36.4 ± 0.2		0.053 ± 0.002						
S-TiO_2_-5%	PVDF-g-PSSA	40.9 ± 0.1		0.067 ± 0.002		224.24	130.54		91	
S-TiO_2_-7.5%	PVDF-g-PSSA	32.6 ± 0.2		0.052 ± 0.003						
	Nafion 117	20 ± 0.1		0.078 ± 0.003		210.57	132.02		74	

IA—inorganic additives, WU—water uptake, IEC—ion exchange capacity, PC—proton conductivity, K0—oxygen mass transfer coefficient, IR—internal resistance, PD—power density, CE—coulombic efficiency.

**Table 3:** Physicochemical properties and performance evaluation results of sulfonated graphene oxide containing polymer membrane for MFC applications.

IA	Polymer	WU (%)	IEC (meqg^−1^)	PC (S/cm)	OCV (mV)	IR (Ω)	CD (mAm^−2^)	PD (mWm^−2^)	CE (%)	COD (%)	Ref.
	Nafion				~716 ± 21			481.3			[150]
	PBI			4.28 × 10^−4^	~477 ± 13		709.19				
SGO-1%	SPBI				~567 ± 16						
SGO-3%	SPBI		0.912		~669 ± 18		2019.06	472.46			
SGO-5%	SPBI	21	1.001	0.018	~625 ± 18		1959.43				
	Nafion	17.5	0.86 ± 0.1	0.0013			233.3 ± 3	35.9 ± 2	2.28 ± 0.2	82.71 ± 5	[154]
	PES	5.41	0.28 ± 0.1	0.00021							
	SPES	12.33	0.36 ± 0.1	0.00032			216.7 ± 3	27.8 ± 2	2.19 ± 0.2	79.76 ± 5	
GO-0.6%	SPES	20.71	0.42 ± 0.1	0.00044			218.7 ± 7	28.5 ± 2	2.04 ± 0.2	75.2 ± 5	
GO-1.2%	SPES	22	0.54 ± 0.1	0.00052			222.1 ± 3	32.1 ± 2	2.20 ± 0.2	80.74 ± 5	
GO-1.8%	SPES	23.13	0.74 ± 0.1	0.00056			225 ± 3±3	39.2 ± 2	2.28 ± 0.2	86.47 ± 5	
SGO-0.6%	SPES	28.41	0.61 ± 0.1	0.00105			233.5 ± 3	46.8 ± 2	2.13 ± 0.2	78.21 ± 5	
SGO-1.2%	SPES	33.54	0.72 ± 0.1	0.00125			276.4 ± 3	61.5 ± 2	2.64 ± 0.2	85.41 ± 5	
SGO-1.8%	SPES	36.32	0.84 ± 0.1	0.00142			300 ± 3	66.4 ± 2	3.73 ± 0.2	89.85 ± 5	
TGO-0.6%	SPES	24.55	0.55 ± 0.1	0.00084			223.6 ± 3	43.4 ± 2	2.18 ± 0.2	72.53 ± 5	
TGO-1.2%	SPES	26.22	0.63 ± 0.1	0.00101			255 ± 3	51.7 ± 2	2.51 ± 0.2	85.22 ± 5	
TGO-1.8%	SPES	30	0.77 ± 0.1	0.00125			275 ± 3	54.13 ± 2	3.20 ± 0.2	88.07 ± 5	
	PVDF-g-PSSA	25.00	0.58	0.046		243.9		106.67			[151]
GO-1.0%	PVDF-g-PSSA	29.32	0.79	0.065		189.		138.02			
SGO-0.1%	PVDF-g-PSSA	26.45	0.64	0.056							
SGO-0.5%	PVDF-g-PSSA	30.14	0.81	0.068							
SGO-1.0%	PVDF-g-PSSA	32.56	1.24	0.083		167.9		180.27			
SGO-1.5%	PVDF-g-PSSA	33.05	1.08	0.079							
SGO-2.0%	PVDF-g-PSSA	34.24	0.85	0.070							
	Nafion	20.00	0.89	0.078		210.5		132.02			
	Nafion	20.1 ± 0.1	1.0 ± 0.1	0.071 ± 0.003		210		132		74	[155]
	PVDF-g-PSSA	25.0 ± 0.2	0.60 ± 0.2	0.046 ± 0.003		228		147		68	
SGO-1.0%	PP	32.4 ± 0.2	1.4 ± 0.2	0.073 ± 0.002		191		166		71	
SGO@SiO_2_-0.1%	PVDF-g-PSSA	26.1 ± 0.3	1.0 ± 0.2	0.068 ± 0.002							
SGO@SiO_2_-0.5%	PVDF-g-PSSA	30.1 ± 0.1	1.2 ± 0.2	0.072 ± 0.004							
SGO@SiO_2_-1.0%	PVDF-g-PSSA	34.2 ± 0.2	1.6 ± 0.1	0.078 ± 0.004		152	1338	185		75	
SGO@SiO_2_-2.0%	PVDF-g-PSSA	33.6 ± 0.2	1.4 ± 0.2	0.074 ± 0.003							

IA—inorganic additives, WU—water uptake, IEC—ion exchange capacity, PC—proton conductivity, OCV—open circuit voltage, IR—internal resistance, CD—current density, PD—power density, CE—coulombic efficiency.

**Table 4:** Physicochemical properties and performance evaluation results of different kinds of sulfonated additives containing polymer membrane for MFC applications.

IA	Polymer	WU (%)	IEC (meqg^−1^)	PC (S/cm)	K0 (cms^−1^)	OCV (mv)	IR (Ω)	CD (mAm^−2^)	PD (mWm^−2^)	Ref.
Fe_3_O_4_-10%	PES	1.56 ± 0.24	0.07 ± 0.02		1.26 × 10^−3^	550.50 ± 3.53	13.87 ± 1.79	20.78 ± 0.30	5.72 ± 0.81	[162]
SFe_3_O_4_-5%	PES	1.99 ± 0.04	2.08 ± 0.19		1.10 × 10^−4^	580.90 ± 32.4	7.92 ± 0.75	47.96 ± 3.58	18.70 ± 0.48	
SFe_3_O_4_-10%	PES	3.78 ± 0.20	5.49 ± 0.43		1.28 × 10^−4^	150.67 ± 8.45	5.77 ± 0.96	150.67 ± 8.4	65.24 ± 5.79	
SFe_3_O_4_-15%	PES	3.98 ± 0.01	6.49 ± 0.19		1.45 × 10^−4^	132.84 ± 4.29	5.42 ± 0.25	132.84 ± 4.2	47.82 ± 4.32	
SFe_3_O_4_-20%	PES	5.23 ± 0.30	9.76 ± 0.52		1.46 × 10^−4^	104.91 ± 5.62	5.91 ± 0.33	104.91 ± 5.6	32.52 ± 3.34	
	SPSEBS	37.9	1.9	0.98 × 10^−2^	2.2 × 10^−6^		42			[163]
SPOSS-2%	SPSEBS	38.2	2.6	1.2 × 10^−2^	1.7 × 10^−6^		38			
SPOSS-4%	SPSEBS	39.9	2.9	1.36 × 10^−2^	1.9 × 10^−6^		36			
SPOSS-6%	SPSEBS	41	3.7	1.40 × 10^−2^	1.6 × 10^−6^		31		126	
SPOSS-8%	SPSEBS	39.4	2.9	1.3 × 10^−2^	1.9 × 10^−6^		35			
	Nafion	22	1.2	0.77 × 10^−2^	2.4 × 10^−6^		45			
	SPEEK	29 ± 0.05	1.4 ± 0.3	0.92 × 10^−2^	2.1 × 10^−6^		37		100 ± 1.2	[164]
S-POSS-2.5%	SPEEK	33.8 ± 0.03	1.5 ± 0.06	1.25 × 10^−2^	1.65 × 10^−6^		31			
S-POSS-5%	SPEEK	35.5 ± 0.08	1.8 ± 0.05	1.31 × 10^−2^	1.42 × 10^−6^		28		162 ± 1.4	
S-POSS-7.5%	SPEEK	34.6 ± 0.07	1.7 ± 0.02	1.12 × 10^−2^	1.69 × 10^−6^		33			
S-POSS-10%	SPEEK	32.4 ± 0.02	1.6 ± 0.07	1.02 × 10^−2^	1.82 × 10^−6^		35			
	Nafion	22 ± 0.03	1.2 ± 0.05	0.81 × 10^−2^	2.4 × 10^−6^		40		154 ± 1.7	
	SPEES	29.8 ± 0.3	1.4 ± 0.05	1.12 × 10^−2^	1.9 × 10^−6^		36		75	[165]
ZnO-7.5%	SPEES	30.5 ± 0.2	1.3 ± 0.05	1.19 × 10^−2^	1.83 × 10^−6^		34			
S-ZnO NR-2.5%	SPEES	32.5 ± 0.2	1.6 ± 0.03	1.29 × 10^−2^	1.71 × 10^−6^		32			
S-ZnO NR-5%	SPEES	33.7 ± 0.5	1.8 ± 0.07	1.36 × 10^−2^	1.67 × 10^−6^		30			
S-ZnO NR-7.5%	SPEES	34.6 ± 0.6	2.0 ± 0.05	1.4 × 10^−2^	1.51 × 10^−6^		29		142	
S-ZnO NR-10%	SPEES	31.8 ± 0.9	1.9 ± 0.04	1.21 × 10^−2^	2.45 × 10^−6^		34			
	Nafion	22 ± 0.5	1.2 ± 0.06	0.81 × 10^−2^			40		130	
6% SZnO NR	SPSEBS			1.49 × 10^−2^					147	[166]

IA—inorganic additives, WU—water uptake, IEC—ion exchange capacity, PC—proton conductivity, K0—oxygen mass transfer coefficient, OCV—open circuit voltage, IR—internal resistance, CD—current density, PD—power density.

## Data Availability

Not applicable.

## References

[1] Abdelkareem M.A., Elsaid K., Wilberforce T., Kamil M., Sayed E.T., Olabi A. (2021). Environmental aspects of fuel cells: A review. Sci. Total. Environ..

[2] Sazali N., Wan Salleh W.N., Jamaludin A.S., Mhd Razali M.N. (2020). New Perspectives on Fuel Cell Technology: A Brief Review. Membranes.

[3] Olabi A., Wilberforce T., Abdelkareem M.A. (2021). Fuel cell application in the automotive industry and future perspective. Energy.

[4] Stambouli A.B., Traversa E. (2002). Fuel cells, an alternative to standard sources of energy. Renew. Sustain. Energy Rev..

[5] Ang T.-Z., Salem M., Kamarol M., Das H.S., Nazari M.A., Prabaharan N. (2022). A comprehensive study of renewable energy sources: Classifications, challenges and suggestions. Energy Strat. Rev..

[6] Zhao J., Patwary A.K., Qayyum A., Alharthi M., Bashir F., Mohsin M., Hanif I., Abbas Q. (2022). The determinants of renewable energy sources for the fueling of green and sustainable economy. Energy.

[7] Sadhasivam T., Jung H.Y., Pandikumar A., Rameshkumar P. (2020). Chapter 3—Nanostructured bifunctional electrocatalyst support materials for unitized regenerative fuel cells. Nanostructured, Functional, and Flexible Materials for Energy Conversion and Storage Systems.

[8] Thangarasu S., Oh T.H. (2021). Progress in poly(phenylene oxide) based cation exchange membranes for fuel cells and redox flow batteries applications. Int. J. Hydrogen Energy.

[9] Thangarasu S., Oh T.-H. (2022). Recent Developments on Bioinspired Cellulose Containing Polymer Nanocomposite Cation and Anion Exchange Membranes for Fuel Cells (PEMFC and AFC). Polymers.

[10] Roh S.-H., Palanisamy G., Sadhasivam T., Jin J.-E., Shim J.-Y., Jung H.-Y. (2019). Techno-Economical Feasibility of Biocellulose Membrane along with Polyethylene Film as a Separator for Lead-Acid Batteries. ACS Sustain. Chem. Eng..

[11] Palanisamy G., Sadhasivam T., Park W.-S., Bae S.T., Roh S.-H., Jung H.-Y. (2020). Tuning the Ion Selectivity and Chemical Stability of a Biocellulose Membrane by PFSA Ionomer Reinforcement for Vanadium Redox Flow Battery Applications. ACS Sustain. Chem. Eng..

[12] Magdum S.S., Thangarasu S., Oh T.H. (2022). Three-Dimensional Ternary rGO/VS2/WS2 Composite Hydrogel for Supercapacitor Applications. Inorganics.

[13] Lin T.W., Sadhasivam T., Wang A.Y., Chen T.Y., Lin J.Y., Shao L.D. (2018). Ternary Composite Nanosheets with MoS2/WS2/Graphene Heterostructures as High-Performance Cathode Materials for Supercapacitors. ChemElectroChem.

[14] Winter M., Brodd R.J. (2004). What Are Batteries, Fuel Cells, and Supercapacitors?. Chem. Rev..

[15] Cyril P.H., Saravanan G. (2020). Development of advanced materials for cleaner energy generation through fuel cells. N. J. Chem..

[16] Fan L., Tu Z., Chan S.H. (2021). Recent development of hydrogen and fuel cell technologies: A review. Energy Rep..

[17] Golubenko D.V., Korchagin O.V., Voropaeva D.Y., Bogdanovskaya V.A., Yaroslavtsev A.B. (2022). Membranes Based on Polyvinylidene Fluoride and Radiation-Grafted Sulfonated Polystyrene and Their Performance in Proton-Exchange Membrane Fuel Cells. Polymers.

[18] Samsudin A.M., Bodner M., Hacker V. (2022). A Brief Review of Poly(Vinyl Alcohol)-Based Anion Exchange Membranes for Alkaline Fuel Cells. Polymers.

[19] Wang R.-T., Chang H.-Y., Wang J.-C. (2021). An Overview on the Novel Core-Shell Electrodes for Solid Oxide Fuel Cell (SOFC) Using Polymeric Methodology. Polymers.

[20] Jang I., Ahn M., Lee S., Yoo S.J. (2022). Surfactant assisted geometric barriers on PtNi@C electrocatalyst for phosphoric acid fuel cells. J. Ind. Eng. Chem..

[21] Luo J., Tian W., Jin H., Yang J., Li J., Wang Y., Shen W., Ren Y., Zhou M. (2023). Recent advances in microbial fuel cells: A review on the identification technology, molecular tool and improvement strategy of electricigens. Curr. Opin. Electrochem..

[22] Ma J., Zhang J., Zhang Y., Guo Q., Hu T., Xiao H., Lu W., Jia J. (2023). Progress on anodic modification materials and future development directions in microbial fuel cells. J. Power Sources.

[23] Palanisamy G., Thangarasu S., Dharman R.K., Patil C.S., Negi T.P.P.S., Kurkuri M.D., Pai R.K., Oh T.H. (2023). The growth of biopolymers and natural earthen sources as membrane/separator materials for microbial fuel cells: A comprehensive review. J. Energy Chem..

[24] Pasquini L., Zhakisheva B., Sgreccia E., Narducci R., Di Vona M., Knauth P. (2021). Stability of Proton Exchange Membranes in Phosphate Buffer for Enzymatic Fuel Cell Application: Hydration, Conductivity and Mechanical Properties. Polymers.

[25] Palanisamy G., Oh T.H., Thangarasu S. (2023). Modified Cellulose Proton-Exchange Membranes for Direct Methanol Fuel Cells. Polymers.

[26] Milewski J., Zdeb J., Szczęśniak A., Martsinchyk A., Kupecki J., Dybiński O. (2023). Concept of a solid oxide electrolysis-molten carbonate fuel cell hybrid system to support a power-to-gas installation. Energy Convers. Manag..

[27] Saritha D., Reddy N.M., Ramesh G.V. (2022). Pt- and Pd- based intermetallic anode catalysts for direct ethanol fuel cell (DEFC): An overview. Mater. Today Proc..

[28] Thangarasu S., Jung H.-Y., Wee J.-H., Kim Y.A., Roh S.-H. (2021). A new strategy of carbon—Pb composite as a bipolar plate material for unitized regenerative fuel cell system. Electrochim. Acta.

[29] SRoh S.-H., Sadhasivam T., Kim H., Park J.-H., Jung H.-Y. (2016). Carbon free SiO2–SO3H supported Pt bifunctional electrocatalyst for unitized regenerative fuel cells. Int. J. Hydrogen Energy.

[30] Gouda M.H., Elessawy N.A., Al-Hussain S.A., Toghan A. (2021). Design of Promising Green Cation-Exchange-Membranes-Based Sulfonated PVA and Doped with Nano Sulfated Zirconia for Direct Borohydride Fuel Cells. Polymers.

[31] Cheng Y., Sun Y., Deng X., Zhang M., Zhang L., Wang W. (2022). High-performance high-entropy quinary-alloys as anode catalysts for direct ethylene glycol fuel cells. Int. J. Hydrogen Energy.

[32] Banjong J., Therdthianwong A., Therdthianwong S., Yongprapat S., Wongyao N. (2020). High performance alkaline-acid direct glycerol fuel cells for portable power supplies via electrode structure design. Int. J. Hydrogen Energy.

[33] Jałowiecka M., Bojarska Z., Małolepszy A., Makowski Ł. (2023). Mass transport enhancement in direct formic acid fuel cell with a novel channel design. Chem. Eng. J..

[34] Palanisamy G., Jung H.-Y., Sadhasivam T., Kurkuri M.D., Kim S.C., Roh S.-H. (2019). A comprehensive review on microbial fuel cell technologies: Processes, utilization, and advanced developments in electrodes and membranes. J. Clean. Prod..

[35] Mukimin A., Vistanty H. (2023). Low carbon development based on microbial fuel cells as electrical generation and wastewater treatment unit. Renew. Energy Focus.

[36] Sonawane J.M., Mahadevan R., Pandey A., Greener J. (2022). Recent progress in microbial fuel cells using substrates from diverse sources. Heliyon.

[37] Bhagat M.S., Mungray A.K., Mungray A.A. (2022). Mungray, Recent advances in osmotic microbial fuel cell technology: A review. J. Indian Chem. Soc..

[38] Barakat N.A.M., Amen M.T., Ali R.H., Nassar M.M., Fadali O.A., Ali M.A., Kim H.Y. (2022). Carbon Nanofiber Double Active Layer and Co-Incorporation as New Anode Modification Strategies for Power-Enhanced Microbial Fuel Cells. Polymers.

[39] Yaqoob A.A., Serrà A., Bhawani S.A., Ibrahim M.N.M., Khan A., Alorfi H.S., Asiri A.M., Hussein M.A., Khan I., Umar K. (2022). Utilizing Biomass-Based Graphene Oxide–Polyaniline–Ag Electrodes in Microbial Fuel Cells to Boost Energy Generation and Heavy Metal Removal. Polymers.

[40] Erensoy A., Çek N. (2021). Investigation of Polymer Biofilm Formation on Titanium-Based Anode Surface in Microbial Fuel Cells with Poplar Substrate. Polymers.

[41] WXue W., Chanamarn W., Tabucanon A.S., Cruz S.G., Hu Y. (2022). Treatment of agro-food industrial waste streams using osmotic microbial fuel cells: Performance and potential improvement measures. Environ. Technol. Innov..

[42] Sahu O. (2019). Sustainable and clean treatment of industrial wastewater with microbial fuel cell. Results Eng..

[43] Gude V.G. (2016). Wastewater treatment in microbial fuel cells—An overview. J. Clean. Prod..

[44] Moqsud M.A., Omine K., Yasufuku N., Hyodo M., Nakata Y. (2013). Microbial fuel cell (MFC) for bioelectricity generation from organic wastes. Waste Manag..

[45] Rojas-Flores S., Benites S., De La Cruz-Noriega M., Cabanillas-Chirinos L., Valdiviezo-Dominguez F., Álvarez M.Q., Vega-Ybañez V., Angelats-Silva L. (2021). Bioelectricity Production from Blueberry Waste. Processes.

[46] Naseer M.N., Zaidi A.A., Khan H., Kumar S., bin Owais M.T., Jaafar J., Suhaimin N.S., Wahab Y.A., Dutta K., Asif M. (2021). Mapping the field of microbial fuel cell: A quantitative literature review (1970–2020). Energy Rep..

[47] Wang J., Ren K., Zhu Y., Huang J., Liu S. (2022). A Review of Recent Advances in Microbial Fuel Cells: Preparation, Operation, and Application. Biotech.

[48] Wang D., Wang Y., Yang J., He X., Wang R.-J., Lu Z.-S., Qiao Y. (2020). Cellulose Aerogel Derived Hierarchical Porous Carbon for Enhancing Flavin-Based Interfacial Electron Transfer in Microbial Fuel Cells. Polymers.

[49] Wu W., Niu H., Yang D., Wang S., Jiang N., Wang J., Lin J., Hu C. (2018). Polyaniline/Carbon Nanotubes Composite Modified Anode via Graft Polymerization and Self-Assembling for Microbial Fuel Cells. Polymers.

[50] Saravanan A., Karishma S., Kumar P.S., Yaashikaa P.R., Jeevanantham S., Gayathri B. (2020). Microbial electrolysis cells and microbial fuel cells for biohydrogen production: Current advances and emerging challenges. Biomass-Convers. Biorefinery.

[51] Ahmed S.F., Mofijur M., Islam N., Parisa T.A., Rafa N., Bokhari A., Klemeš J.J., Mahlia T.M.I. (2022). Insights into the development of microbial fuel cells for generating biohydrogen, bioelectricity, and treating wastewater. Energy.

[52] Thulasinathan B., Jayabalan T., Arumugam N., Kulanthaisamy M.R., Kim W., Kumar P., Govarthanan M., Alagarsamy A. (2022). Wastewater substrates in microbial fuel cell systems for carbon-neutral bioelectricity generation: An overview. Fuel.

[53] Ren H., Lee H.-S., Chae J. (2012). Miniaturizing microbial fuel cells for potential portable power sources: Promises and challenges. Microfluid. Nanofluidics.

[54] Mateo S., Mascia M., Fernandez-Morales F.J., Rodrigo M.A., Di Lorenzo M. (2019). Assessing the impact of design factors on the performance of two miniature microbial fuel cells. Electrochim. Acta.

[55] Kumar R., Singh L., Zularisam A.W., Singh L., Kalia V.C. (2017). Microbial Fuel Cells: Types and Applications. Waste Biomass Management—A Holistic Approach.

[56] Logan B.E., Hamelers B., Rozendal R., Schröder U., Keller J., Freguia S., Aelterman P., Verstraete W., Rabaey K. (2006). Microbial Fuel Cells:  Methodology and Technology. Environ. Sci. Technol..

[57] Rojas-Flores S., De La Cruz-Noriega M., Nazario-Naveda R., Benites S.M., Delfín-Narciso D., Rojas-Villacorta W., Romero C.V. (2022). Bioelectricity through microbial fuel cells using avocado waste. Energy Rep..

[58] Aiswaria P., Mohamed S.N., Singaravelu D.L., Brindhadevi K., Pugazhendhi A. (2022). A review on graphene/graphene oxide supported electrodes for microbial fuel cell applications: Challenges and prospects. Chemosphere.

[59] ANawaz A., Haq I.U., Qaisar K., Gunes B., Raja S.I., Mohyuddin K., Amin H. (2022). Microbial fuel cells: Insight into simultaneous wastewater treatment and bioelectricity generation. Process. Saf. Environ. Prot..

[60] Roshanravan B., Younesi H., Abdollahi M., Rahimnejad M., Pyo S.-H. (2021). Application of proton-conducting sulfonated polysulfone incorporated MIL-100(Fe) composite materials for polymer-electrolyte membrane microbial fuel cells. J. Clean. Prod..

[61] Sharif H.M.A., Farooq M., Hussain I., Ali M., Mujtaba M., Sultan M., Yang B. (2021). Recent innovations for scaling up microbial fuel cell systems: Significance of physicochemical factors for electrodes and membranes materials. J. Taiwan Inst. Chem. Eng..

[62] Souza L., Antônio R., Hotza D., Carminatti C., Pineda-Vásquez T., Watzko E., Pezzin A.P., Duarte D., Recouvreux D. (2023). Lignin-incorporated bacterial nanocellulose for proton exchange membranes in microbial fuel cells. Mater. Chem. Phys..

[63] Nayak J.K., Shankar U., Samal K. (2023). Fabrication and development of SPEEK/PVdF-HFP/SiO2 proton exchange membrane for microbial fuel cell application. Chem. Eng. J. Adv..

[64] Terbish N., Popuri S.R., Lee C.-H. (2023). Improved performance of organic–inorganic nanocomposite membrane for bioelectricity generation and wastewater treatment in microbial fuel cells. Fuel.

[65] Ramirez-Nava J., Martínez-Castrejón M., García-Mesino R.L., López-Díaz J.A., Talavera-Mendoza O., Sarmiento-Villagrana A., Rojano F., Hernández-Flores G. (2021). The Implications of Membranes Used as Separators in Microbial Fuel Cells. Membranes.

[66] Ramírez-Carmona M., Gálvez-Gómez M.P., González-Perez L., Pinedo-Rangel V., Pineda-Vasquez T., Hotza D. (2023). Production of Bacterial Cellulose Hydrogel and its Evaluation as a Proton Exchange Membrane. J. Polym. Environ..

[67] Uddin M.J., Jeong Y.K., Lee W. (2021). Microbial fuel cells for bioelectricity generation through reduction of hexavalent chromium in wastewater: A review. Int. J. Hydrogen Energy.

[68] Rahman W., Yusup S., Mohammad S.N.A.A. (2021). Mohammad, Screening of fruit waste as substrate for microbial fuel cell (MFC). AIP Conf. Proc..

[69] Din M.I., Ahmed M., Ahmad M., Iqbal M., Ahmad Z., Hussain Z., Khalid R., Samad A. (2023). Investigating the Activity of Carbon Fiber Electrode for Electricity Generation from Waste Potatoes in a Single-Chambered Microbial Fuel Cell. J. Chem..

[70] Muthukrishnan L., Kamaraj S.K., Sánchez-Olmos L.A., Cardenas M.S., Caballero-Briones F., Jadhav D.A., Pandit S., Gajalakshmi S., Shah M.P. (2022). Chapter 24—Toward sustainable feasibility of microbial electrochemical systems to reality. Scaling Up of Microbial Electrochemical Systems.

[71] Rismani-Yazdi H., Carver S.M., Christy A.D., Tuovinen O.H. (2008). Cathodic limitations in microbial fuel cells: An overview. J. Power Sources.

[72] Ghosh S., Das S., Mosquera M.E.G. (2020). Conducting Polymer-Based Nanohybrids for Fuel Cell Application. Polymers.

[73] Abdallah M., Feroz S., Alani S., Sayed E.T., Shanableh A. (2019). Continuous and scalable applications of microbial fuel cells: A critical review. Rev. Environ. Sci. Bio/Technology.

[74] Tofighi A., Rahimnejad M., Ismail A.F., Salleh W.N.W., Yusof N. (2020). Chapter 14—Synthetic polymer-based membranes for microbial fuel cells. Synthetic Polymeric Membranes for Advanced Water Treatment, Gas Separation, and Energy Sustainability.

[75] Vilela C., Cordeiro D.M., Boas J.V., Barbosa P., Nolasco M., Vaz P.D., Rudić S., Ribeiro-Claro P., Silvestre A.J., Oliveira V.B. (2020). Poly(4-styrene sulfonic acid)/bacterial cellulose membranes: Electrochemical performance in a single-chamber microbial fuel cell. Bioresour. Technol. Rep..

[76] Noori T., Ghangrekar M., Mukherjee C., Min B. (2019). Biofouling effects on the performance of microbial fuel cells and recent advances in biotechnological and chemical strategies for mitigation. Biotechnol. Adv..

[77] Banerjee A., Calay R.K., Eregno F.E. (2022). Role and Important Properties of a Membrane with Its Recent Advancement in a Microbial Fuel Cell. Energies.

[78] Das I., Das S., Dixit R., Ghangrekar M.M. (2020). Goethite supplemented natural clay ceramic as an alternative proton exchange membrane and its application in microbial fuel cell. Ionics.

[79] Koók L., Žitka J., Bakonyi P., Takács P., Pavlovec L., Otmar M., Kurdi R., Bélafi-Bakó K., Nemestóthy N. (2020). Electrochemical and microbiological insights into the use of 1,4-diazabicyclo[2.2.2]octane-functionalized anion exchange membrane in microbial fuel cell: A benchmarking study with Nafion. Sep. Purif. Technol..

[80] Subhadarshini S., Sravan J.S., Sarkar O., Mohan S.V., Roy T.K., Jana T. (2023). Sulfonated Polybenzimidazole as a PEM in a Microbial Fuel Cell: An Efficient Strategy for Green Energy Generation and Wastewater Cleaning. ACS Appl. Energy Mater..

[81] Di Palma L., Bavasso I., Sarasini F., Tirillò J., Puglia D., Dominici F., Torre L. (2018). Synthesis, characterization and performance evaluation of Fe3O4/PES nano composite membranes for microbial fuel cell. Eur. Polym. J..

[82] Rozendal R.A., Hamelers H.V.M., Buisman C.J.N. (2006). Effects of Membrane Cation Transport on pH and Microbial Fuel Cell Performance. Environ. Sci. Technol..

[83] Koók L., Kaufer B., Bakonyi P., Rózsenberszki T., Rivera I., Buitrón G., Bélafi-Bakó K., Nemestóthy N. (2019). Supported ionic liquid membrane based on [bmim][PF6] can be a promising separator to replace Nafion in microbial fuel cells and improve energy recovery: A comparative process evaluation. J. Membr. Sci..

[84] Lim S.S., Daud W.R.W., Jahim J.M., Ghasemi M., Chong P.S., Ismail M. (2012). Sulfonated poly(ether ether ketone)/poly(ether sulfone) composite membranes as an alternative proton exchange membrane in microbial fuel cells. Int. J. Hydrogen Energy.

[85] Rahimnejad M., Bakeri G., Ghasemi M., Zirepour A. (2014). A review on the role of proton exchange membrane on the performance of microbial fuel cell. Polym. Adv. Technol..

[86] Nagar H., Badhrachalam N., Rao V.B., Sridhar S. (2019). A novel microbial fuel cell incorporated with polyvinylchloride/4A zeolite composite membrane for kitchen wastewater reclamation and power generation. Mater. Chem. Phys..

[87] Zinadini S., Zinatizadeh A., Rahimi M., Vatanpour V., Rahimi Z. (2017). High power generation and COD removal in a microbial fuel cell operated by a novel sulfonated PES/PES blend proton exchange membrane. Energy.

[88] Singha S., Jana T., Modestra J.A., Kumar A.N., Mohan S.V. (2016). Highly efficient sulfonated polybenzimidazole as a proton exchange membrane for microbial fuel cells. J. Power Sources.

[89] Kumar V., Mondal S., Nandy A., Kundu P.P. (2016). Analysis of polybenzimidazole and polyvinylpyrrolidone blend membranes as separating barrier in single chambered microbial fuel cells. Biochem. Eng. J..

[90] Kamaraj S.-K., Romano S.M., Moreno V.C., Poggi-Varaldo H., Solorza-Feria O. (2015). Use of Novel Reinforced Cation Exchange Membranes for Microbial Fuel Cells. Electrochim. Acta.

[91] Rudra R., Kumar V., Kundu P.P. (2015). Acid catalysed cross-linking of poly vinyl alcohol (PVA) by glutaraldehyde: Effect of crosslink density on the characteristics of PVA membranes used in single chambered microbial fuel cells. RSC Adv..

[92] Terbish N., Lee C.-H., Popuri S.R., Nalluri L.P. (2020). An investigation into polymer blending, plasticization and cross-linking effect on the performance of chitosan-based composite proton exchange membranes for microbial fuel cell applications. J. Polym. Res..

[93] Ying Y., Kamarudin S., Masdar M. (2018). Silica-related membranes in fuel cell applications: An overview. Int. J. Hydrogen Energy.

[94] Zhang H., Shen P.K. (2012). Recent Development of Polymer Electrolyte Membranes for Fuel Cells. Chem. Rev..

[95] Salarizadeh P., Javanbakht M., Pourmahdian S., Hazer M.S.A., Hooshyari K., Askari M.B. (2019). Novel proton exchange membranes based on proton conductive sulfonated PAMPS/PSSA-TiO2 hybrid nanoparticles and sulfonated poly (ether ether ketone) for PEMFC. Int. J. Hydrogen Energy.

[96] Tripathi B.P., Shahi V.K. (2011). Organic–inorganic nanocomposite polymer electrolyte membranes for fuel cell applications. Prog. Polym. Sci..

[97] Guo Z., Chen J., Byun J.J., Cai R., Perez-Page M., Sahoo M., Ji Z., Haigh S.J., Holmes S.M. (2022). High-performance polymer electrolyte membranes incorporated with 2D silica nanosheets in high-temperature proton exchange membrane fuel cells. J. Energy Chem..

[98] Yoonoo C., Dawson C.P., Roberts E.P., Holmes S.M. (2011). Nafion®/mordenite composite membranes for improved direct methanol fuel cell performance. J. Membr. Sci..

[99] Al-Batty S., Dawson C., Shanmukham S.P., Roberts E.P.L., Holmes S.M. (2016). Improvement of direct methanol fuel cell performance using a novel mordenite barrier layer. J. Mater. Chem. A.

[100] Xi J., Wu Z., Qiu X., Chen L. (2007). Nafion/SiO2 hybrid membrane for vanadium redox flow battery. J. Power Sources.

[101] Sadhasivam T., Kim H.-T., Park W.-S., Lim H., Ryi S.-K., Roh S.-H., Jung H.-Y. (2017). Low permeable composite membrane based on sulfonated poly(phenylene oxide) (sPPO) and silica for vanadium redox flow battery. Int. J. Hydrogen Energy.

[102] Kou W., Li X., Liu Y., Zhang X., Yang S., Jiang X., He G., Dai Y., Zheng W., Yu G. (2019). Triple-Layered Carbon-SiO2 Composite Membrane for High Energy Density and Long Cycling Li–S Batteries. ACS Nano.

[103] Bisht S., Balaguru S., Ramachandran S.K., Gangasalam A., Kweon J. (2021). Proton exchange composite membranes comprising SiO2, sulfonated SiO2, and metal–organic frameworks loaded in SPEEK polymer for fuel cell applications. J. Appl. Polym. Sci..

[104] Sivasankaran A., Sangeetha D., Ahn Y.-H. (2016). Nanocomposite membranes based on sulfonated polystyrene ethylene butylene polystyrene (SSEBS) and sulfonated SiO2 for microbial fuel cell application. Chem. Eng. J..

[105] Sivasankaran A., Sangeetha D. (2015). Influence of sulfonated SiO2 in sulfonated polyether ether ketone nanocomposite membrane in microbial fuel cell. Fuel.

[106] Kircheva N., Outin J., Perrier G., Ramousse J., Merlin G., Lyautey E. (2015). Bio-electrochemical characterization of air-cathode microbial fuel cells with microporous polyethylene/silica membrane as separator. Bioelectrochemistry.

[107] Gaurav K., Singh R., Tiwari B.K., Srivastava R. (2019). Novel proton exchange membranes based on PVC for microbial fuel cells (MFCs). J. Polym. Eng..

[108] Angioni S., Millia L., Bruni G., Tealdi C., Mustarelli P., Quartarone E. (2016). Quartarone, Improving the performances of Nafion™-based membranes for microbial fuel cells with silica-based, organically-functionalized mesostructured fillers. J. Power Sources.

[109] Bi C., Zhang H., Zhang Y., Zhu X., Ma Y., Dai H., Xiao S. (2008). Fabrication and investigation of SiO2 supported sulfated zirconia/Nafion® self-humidifying membrane for proton exchange membrane fuel cell applications. J. Power Sources.

[110] Nunes S.P., Ruffmann B., Rikowski E., Vetter S., Richau K. (2002). Inorganic modification of proton conductive polymer membranes for direct methanol fuel cells. J. Membr. Sci..

[111] Kugarajah V., Sugumar M., Dharmalingam S. (2020). Nanocomposite membrane and microbial community analysis for improved performance in microbial fuel cell. Enzym. Microb. Technol..

[112] Bajestani M.B., Mousavi S.A. (2016). Effect of casting solvent on the characteristics of Nafion/TiO2 nanocomposite membranes for microbial fuel cell application. Int. J. Hydrogen Energy.

[113] Kim S.H., Kwak S.-Y., Sohn B.-H., Park T.H. (2003). Design of TiO2 nanoparticle self-assembled aromatic polyamide thin-film-composite (TFC) membrane as an approach to solve biofouling problem. J. Membr. Sci..

[114] Bae T.-H., Tak T.-M. (2005). Effect of TiO2 nanoparticles on fouling mitigation of ultrafiltration membranes for activated sludge filtration. J. Membr. Sci..

[115] Pagnout C., Jomini S., Dadhwal M., Caillet C., Thomas F., Bauda P. (2012). Role of electrostatic interactions in the toxicity of titanium dioxide nanoparticles toward Escherichia coli. Colloids Surf. B Biointerfaces.

[116] Martinez-Morlanes M.J., De La Torre-Gamarra C., Pérez-Prior M.T., Lara-Benito S., Del Rio C., Várez A., Levenfeld B. (2021). Sulfonated Polysulfone/TiO2(B) Nanowires Composite Membranes as Polymer Electrolytes in Fuel Cells. Polymers.

[117] Kim A.R., Vinothkannan M., Lee K.H., Chu J.Y., Park B., Han M., Yoo D.J. (2022). Enhanced performance and durability of composite membranes containing anatase titanium oxide for fuel cells operating under low relative humidity. Int. J. Energy Res..

[118] Kumar K.S., Prabhu M.R. (2021). Enhancing Proton Conduction of Poly(benzimidazole) with Sulfonated Titania Nano Composite Membrane for PEM Fuel Cell Applications. Macromol. Res..

[119] Palanisamy G., Oh T.H. (2022). TiO2 Containing Hybrid Composite Polymer Membranes for Vanadium Redox Flow Batteries. Polymers.

[120] Wang G., Wang F., Li A., Zhang M., Zhang J., Chen J., Wang R. (2020). Sulfonated poly(ether ether ketone)/s–TiO2 composite membrane for a vanadium redox flow battery. J. Appl. Polym. Sci..

[121] Harun N.A.M., Shaari N., Zaiman N.F.H.N. (2021). A review of alternative polymer electrolyte membrane for fuel cell application based on sulfonated poly(ether ether ketone). Int. J. Energy Res..

[122] Wu J., Nie S., Liu H., Gong C., Zhang Q., Xu Z., Liao G. (2022). Design and development of nucleobase modified sulfonated poly(ether ether ketone) membranes for high-performance direct methanol fuel cells. J. Mater. Chem. A.

[123] Mahimai B.M., Sivasubramanian G., Sekar K., Kannaiyan D., Deivanayagam P. (2022). Sulfonated poly(ether ether ketone): Efficient ion-exchange polymer electrolytes for fuel cell applications—A versatile review. Mater. Adv..

[124] Venkatesan P.N., Dharmalingam S. (2015). Effect of cation transport of SPEEK—Rutile TiO2 electrolyte on microbial fuel cell performance. J. Membr. Sci..

[125] Vidhyeswari D., Surendhar A., Bhuvaneshwari S. (2021). Evaluation of power generation and treatment efficiency of dairy wastewater in microbial fuel cell using TiO2—SPEEK as proton exchange membrane. Water Sci. Technol..

[126] Bhowmick G.D., Dhar D., Ghangrekar M.M., Banerjee R. (2020). TiO2-Si- or SrTiO3-Si-impregnated PVA–based low-cost proton exchange membranes for application in microbial fuel cell. Ionics.

[127] Martinelli A., Matic A., Jacobsson P., Börjesson L., Navarra M., Fernicola A., Panero S., Scrosati B. (2006). Structural analysis of PVA-based proton conducting membranes. Solid State Ion..

[128] Münch W., Kreuer K.D., Seifert G., Maier J. (2000). Proton diffusion in perovskites: Comparison between BaCeO3, BaZrO3, SrTiO3, and CaTiO3 using quantum molecular dynamics. Solid State Ion..

[129] Soria R.B., Chinchin B.D., Arboleda D., Zhao Y., Bonilla P., Van der Bruggen B., Luis P. (2022). Effect of the bio-inspired modification of low-cost membranes with TiO2:ZnO as microbial fuel cell membranes. Chemosphere.

[130] Kugarajah V., Dharmalingam S. (2020). Investigation of a cation exchange membrane comprising Sulphonated Poly Ether Ether Ketone and Sulphonated Titanium Nanotubes in Microbial Fuel Cell and preliminary insights on microbial adhesion. Chem. Eng. J..

[131] Ayyaru S., Dharmalingam S. (2015). A study of influence on nanocomposite membrane of sulfonated TiO2 and sulfonated polystyrene-ethylene-butylene-polystyrene for microbial fuel cell application. Energy.

[132] Pal A., Dey T.K., Debnath A.K., Bhushan B., Sahu A.K., Bindal R.C., Kar S. (2017). Mixed-matrix membranes with enhanced antifouling activity: Probing the surface-tailoring potential of Tiron and chromotropic acid for nano-TiO2. R. Soc. Open Sci..

[133] Ayyaru S., Dharmalingam S. (2013). Improved performance of microbial fuel cells using sulfonated polyether ether ketone (SPEEK) TiO2–SO3H nanocomposite membrane. RSC Adv..

[134] Sugumar M., Dharmalingam S. (2022). Statistical assessment of operational parameters using optimized sulphonated titanium nanotubes incorporated sulphonated polystyrene ethylene butylene polystyrene nanocomposite membrane for efficient electricity generation in microbial fuel cell. Energy.

[135] Kumar V., Rudra R., Hait S. (2021). Sulfonated polyvinylidene fluoride-crosslinked-aniline-2-sulfonic acid as ion exchange membrane in single-chambered microbial fuel cell. J. Environ. Chem. Eng..

[136] Jung H.-Y., Roh S.-H. (2020). Polyvinylidene Fluoride Nanofiber Composite Membrane Coated with Perfluorinated Sulfuric Acid for Microbial Fuel Cell Application. J. Nanosci. Nanotechnol..

[137] Sharma M., Das P.P., Sood T., Chakraborty A., Purkait M.K. (2022). Reduced graphene oxide incorporated polyvinylidene fluoride/cellulose acetate proton exchange membrane for energy extraction using microbial fuel cells. J. Electroanal. Chem..

[138] Li C., Wang L., Wang X., Kong M., Zhang Q., Li G. (2017). Synthesis of PVDF-g-PSSA proton exchange membrane by ozone-induced graft copolymerization and its application in microbial fuel cells. J. Membr. Sci..

[139] Li C., Song Y., Wang X., Zhang Q. (2020). Synthesis, characterization and application of S-TiO2/PVDF-g-PSSA composite membrane for improved performance in MFCs. Fuel.

[140] Leong J.X., Daud W.R.W., Ghasemi M., Ahmad A., Ismail M., Ben Liew K. (2015). Composite membrane containing graphene oxide in sulfonated polyether ether ketone in microbial fuel cell applications. Int. J. Hydrogen Energy.

[141] Cao Y.C., Xu C., Wu X., Wang X., Xing L., Scott K. (2011). A poly (ethylene oxide)/graphene oxide electrolyte membrane for low temperature polymer fuel cells. J. Power Sources.

[142] Chien H.-C., Tsai L.-D., Huang C.-P., Kang C.-Y., Lin J.-N., Chang F.-C. (2013). Sulfonated graphene oxide/Nafion composite membranes for high-performance direct methanol fuel cells. Int. J. Hydrogen Energy.

[143] Shabani M., Younesi H., Rahimpour A., Rahimnejad M. (2019). Upgrading the electrochemical performance of graphene oxide-blended sulfonated polyetheretherketone composite polymer electrolyte membrane for microbial fuel cell application. Biocatal. Agric. Biotechnol..

[144] Ben Liew K., Leong J.X., Daud W.R.W., Ahmad A., Hwang J.J., Wu W. (2020). Incorporation of silver graphene oxide and graphene oxide nanoparticles in sulfonated polyether ether ketone membrane for power generation in microbial fuel cell. J. Power Sources.

[145] Ali A.K., Ali M.E., Younes A.A., El Fadl M.M.A., Farag A. (2021). Proton exchange membrane based on graphene oxide/polysulfone hybrid nano-composite for simultaneous generation of electricity and wastewater treatment. J. Hazard. Mater..

[146] Khilari S., Pandit S., Ghangrekar M.M., Pradhan D., Das D. (2013). Graphene Oxide-Impregnated PVA–STA Composite Polymer Electrolyte Membrane Separator for Power Generation in a Single-Chambered Microbial Fuel Cell. Ind. Eng. Chem. Res..

[147] Rudra R., Kumar V., Pramanik N., Kundu P.P. (2017). Graphite oxide incorporated crosslinked polyvinyl alcohol and sulfonated styrene nanocomposite membrane as separating barrier in single chambered microbial fuel cell. J. Power Sources.

[148] Sharma M., Das P.P., Sood T., Chakraborty A., Purkait M.K. (2021). Ameliorated polyvinylidene fluoride based proton exchange membrane impregnated with graphene oxide, and cellulose acetate obtained from sugarcane bagasse for application in microbial fuel cell. J. Environ. Chem. Eng..

[149] Holder S.L., Lee C.-H., Popuri S.R. (2017). Simultaneous wastewater treatment and bioelectricity production in microbial fuel cells using cross-linked chitosan-graphene oxide mixed-matrix membranes. Environ. Sci. Pollut. Res..

[150] Mondal S., Papiya F., Ash S.N., Kundu P.P. (2021). Composite membrane of sulfonated polybenzimidazole and sulfonated graphene oxide for potential application in microbial fuel cell. J. Environ. Chem. Eng..

[151] Li C., Wang L., Wang X., Li C., Xu Q., Li G. (2019). Fabrication of a SGO/PVDF-g-PSSA composite proton-exchange membrane and its enhanced performance in microbial fuel cells. J. Chem. Technol. Biotechnol..

[152] Ayyaru S., Ahn Y.-H. (2017). Application of sulfonic acid group functionalized graphene oxide to improve hydrophilicity, permeability, and antifouling of PVDF nanocomposite ultrafiltration membranes. J. Membr. Sci..

[153] Angioni S., Millia L., Bruni G., Ravelli D., Mustarelli P., Quartarone E. (2017). Novel composite polybenzimidazole-based proton exchange membranes as efficient and sustainable separators for microbial fuel cells. J. Power Sources.

[154] Shabani M., Younesi H., Pontié M., Rahimpour A., Rahimnejad M., Guo H., Szymczyk A. (2021). Enhancement of microbial fuel cell efficiency by incorporation of graphene oxide and functionalized graphene oxide in sulfonated polyethersulfone membrane. Renew. Energy.

[155] Xu Q., Wang L., Li C., Wang X., Li C., Geng Y. (2019). Study on improvement of the proton conductivity and anti-fouling of proton exchange membrane by doping SGO@SiO2 in microbial fuel cell applications. Int. J. Hydrogen Energy.

[156] Ranjani M., Yoo D.J., Kumar G.G. (2018). Sulfonated Fe3O4@SiO2 nanorods incorporated sPVdF nanocomposite membranes for DMFC applications. J. Membr. Sci..

[157] Nie S., Wu J., Wang L., Cheng F., Sun Z., Chen X., Liu H., Wen S., Gong C. (2023). Hierarchical Fe3O4@LDH-incorporated composite anion exchange membranes for fuel cells based on magnetic field orientation. Surf. Interfaces.

[158] Lo C.-F., Wu J.-F., Li H.-Y., Hung W.-S., Shih C.-M., Hu C.-C., Liu Y.-L., Lue S.J. (2013). Novel polyvinyl alcohol nanocomposites containing carbon nano-tubes with Fe3O4 pendants for alkaline fuel cell applications. J. Membr. Sci..

[159] Prabhu N.V., Sangeetha D. (2014). Characterization and performance study of sulfonated poly ether ether ketone/Fe3O4 nano composite membrane as electrolyte for microbial fuel cell. Chem. Eng. J..

[160] Tjong S. (2006). Structural and mechanical properties of polymer nanocomposites. Mater. Sci. Eng. R Rep..

[161] Bavasso I., Di Palma L., Puglia D., Luzi F., Dominici F., Tirillò J., Sarasini F., Torre L. (2020). Effect of Pretreatment of Nanocomposite PES-Fe3O4 Separator on Microbial Fuel Cells Performance. Polym. Eng. Sci..

[162] Bavasso I., Bracciale M.P., Sbardella F., Puglia D., Dominici F., Torre L., Tirillò J., Sarasini F., De Rosa I.M., Xin W. (2021). Sulfonated Fe3O4/PES nanocomposites as efficient separators in microbial fuel cells. J. Membr. Sci..

[163] Sugumar M., Dharmalingam S. (2020). Statistical optimization of process parameters in microbial fuel cell for enhanced power production using Sulphonated Polyhedral Oligomeric Silsesquioxane dispersed Sulphonated Polystyrene Ethylene Butylene Polystyrene nanocomposite membranes. J. Power Sources.

[164] Kugarajah V., Dharmalingam S. (2020). Sulphonated polyhedral oligomeric silsesquioxane/sulphonated poly ether ether ketone nanocomposite membranes for microbial fuel cell: Insights to the miniatures involved. Chemosphere.

[165] Kugarajah V., Sugumar M., Swaminathan E., Balasubramani N., Dharmalingam S. (2021). Investigation on sulphonated zinc oxide nanorod incorporated sulphonated poly (1,4-phenylene ether ether sulfone) nanocomposite membranes for improved performance of microbial fuel cell. Int. J. Hydrogen Energy.

[166] Sugumar M., Kugarajah V., Dharmalingam S. (2022). Dharmalingam, Optimization of operational factors using statistical design and analysis of nanofiller incorporated polymer electrolyte membrane towards performance enhancement of microbial fuel cell. Process. Saf. Environ. Prot..

[167] Kugarajah V., Dharmalingam S. (2021). Effect of silver incorporated sulphonated poly ether ether ketone membranes on microbial fuel cell performance and microbial community analysis. Chem. Eng. J..

[168] Swamidoss V.F., Bangaru M., Nalathambi G., Sangeetha D., Selvam A.K. (2019). Silver-incorporated poly vinylidene fluoride nanofibers for bacterial filtration. Aerosol Sci. Technol..

[169] Ma M., You S., Gong X., Dai Y., Zou J., Fu H. (2015). Silver/iron oxide/graphitic carbon composites as bacteriostatic catalysts for enhancing oxygen reduction in microbial fuel cells. J. Power Sources.

[170] Davies R.L., Etris S.F. (1997). The development and functions of silver in water purification and disease control. Catal. Today.

[171] Noori M.T., Tiwari B.R., Mukherjee C.K., Ghangrekar M.M. (2018). Enhancing the performance of microbial fuel cell using AgPt bimetallic alloy as cathode catalyst and anti-biofouling agent. Int. J. Hydrogen Energy.

[172] Tiwari B., Noori M., Ghangrekar M. (2016). A novel low cost polyvinyl alcohol-Nafion-borosilicate membrane separator for microbial fuel cell. Mater. Chem. Phys..

[173] Venkatesan P.N., Dharmalingam S. (2017). Characterization and performance study of phase inversed Sulfonated Poly Ether Ether Ketone—Silico tungstic composite membrane as an electrolyte for microbial fuel cell applications. Renew. Energy.

[174] Samaei S.H., Bakeri G., Lashkenari M.S. (2021). Performance of the sulfonated poly ether ether ketone proton exchange membrane modified with sulfonated polystyrene and phosphotungstic acid for microbial fuel cell applications. J. Appl. Polym. Sci..

[175] Saniei N., Ghasemi N., Zinatizadeh A., Zinadini S., Ramezani M., Derakhshan A. (2022). Electricity generation enhancement in microbial fuel cell via employing a new SPEEK based proton exchange membrane modified by goethite nanoparticles functionalized with tannic acid and sulfanilic acid. Environ. Technol. Innov..

[176] Yang W., Chata G., Zhang Y., Peng Y., Lu J.E., Wang N., Mercado R., Li J., Chen S. (2019). Graphene oxide-supported zinc cobalt oxides as effective cathode catalysts for microbial fuel cell: High catalytic activity and inhibition of biofilm formation. Nano Energy.

[177] Muthukumar H., Mohammed S.N., Chandrasekaran N., Sekar A.D., Pugazhendhi A., Matheswaran M. (2019). Effect of iron doped Zinc oxide nanoparticles coating in the anode on current generation in microbial electrochemical cells. Int. J. Hydrogen Energy.

